# Contrasting Treatment- and Farm-Level Metrics of Antimicrobial Use Based on Used Daily Dose vs. Defined Daily Dose for the German Antibiotics Minimization Concept

**DOI:** 10.3389/fvets.2022.913197

**Published:** 2022-06-27

**Authors:** Matthias Flor, Bernd-Alois Tenhagen, Annemarie Käsbohrer

**Affiliations:** ^1^Department Biological Safety, German Federal Institute for Risk Assessment, Berlin, Germany; ^2^Unit of Veterinary Public Health and Epidemiology, Department for Farm Animals and Veterinary Public Health, University of Veterinary Medicine, Vienna, Austria

**Keywords:** livestock, used daily dose (UDD), defined daily dose for animals (DDDvet), animal daily dose, benchmarking, antimicrobial use (AMU), treatment frequency

## Abstract

The German Antibiotics Minimization Concept defines a farm-level benchmarking process based on half-yearly treatment frequencies that applies to six animal populations for fattening: calves (≤ 8 months), cattle (> 8 months), piglets (≤ 30 kg), pigs (> 30 kg), broiler chickens, and turkeys. The treatment frequency defined in the Minimization Concept takes into account the number of animals treated, the treatment duration, and the number of active antimicrobial ingredients, for each individual treatment, and is equivalent to a treatment frequency based on the used daily dose (UDD) and the actual weight of the animals at the time of treatment. With data from the German benchmarking system for the seven half-year periods from the second semester 2014 to the end of 2017, we compared UDD-based metrics of antimicrobial use (AMU) at the treatment and the farm level with metrics based on defined daily doses for animals (DDDvet) and standardized animal weights assumed at the time most likely for treatment. We show the extent to which DDDvet-based metrics would introduce errors into the measurement of AMU at the treatment level and consequently at the farm level. To that end, we introduce the average animal daily dose ratio, an aggregate measure that quantifies how choice of antimicrobial substances, deviations of used doses from recommended doses, of recommended doses from DDDvet values, and of treatment weights from standardized weights, affect a farm's AMU metrics. Our results show that in all animal populations considered benchmarking processes would become less successful at targeting high users. This would be especially true for broiler chickens and turkeys where the relative weight gain during fattening is the largest and overdosing appears to be common practice. Therefore, in AMU monitoring systems with the purpose of benchmarking farms UDD-based metrics are preferable to DDDvet-based metrics.

## Introduction

In order to combat antimicrobial resistance (AMR) which has been recognized as a major global health threat (1, 2) an essential control measure is the monitoring of antimicrobial use (AMU) in food-producing animals combined with the requirement to improve management with frequent antimicrobial treatments (3). Metrics for the quantification of AMU are a topic of international discussions, and different countries and stakeholders have adopted various metrics. These can be classified as weight-based, dose-based, or count-based (4).

In Germany, the 16th amendment to the German Medicinal Products Act entered into force in 2013 (5). It introduced the German Antibiotics Minimization Concept (GAMC) pertaining to six animal populations held for meat production: calves for meat production (from weaning up to the age of 8 months), beef cattle (over the age of 8 months), weaned piglets (from weaning up to a body weight of 30 kg), fattening pigs (weighing over 30 kg), broiler chickens (from the day of hatching), and fattening turkeys (from the day of hatching). For better legibility, we refer to these animal populations throughout the manuscript simply as calves ( ≤ 8 months), cattle (> 8 months), piglets (≤ 30 kg), pigs (> 30 kg), chickens, and turkeys, respectively. The concept established a farm-level benchmarking system based on treatment frequencies per animal day. Farms of the same production type are ranked twice per year according to their individual treatment frequencies. The upper quartile of farms has to develop an action plan to reduce AMU and present it to the authorities.

Farmers holding on average more than 20 calves (≤ 8 months), 20 cattle (> 8 months), 250 piglets (≤ 30 kg), 250 pigs (> 30 kg), 10,000 chickens or 1,000 turkeys, are required to report data on each individual treatment to a central database. This includes the name and total amount of the Veterinary Medicinal Product (VMP) used, the animal population, the number of animals treated, and the treatment duration. For each farm and animal population, the treatment frequency then relates the total number of animal-days treated per half-year period to the average number of animals held in that half-year period. Accordingly, the German AMU benchmarking system can be classified as count-based (4). The German treatment frequency meets the classic definition of an incidence rate denoting the occurrence of an event in a given population at risk within a specific time period (6).

In the field of human medicine, antimicrobial consumption is typically measured using numbers of defined daily doses (DDD) per population (e.g., 1,000 inhabitants or hospital beds) and per day, relying on average adult weight and assumed average maintenance doses per day for a drug used for its main indication in adults (7). Analogously, in veterinary medicine defined daily doses for animals [DDDvet; (8)] per animal population at risk within a specified time period and standardized animal weights may be used to derive DDDvet-based treatment frequencies.

Discrepancies between treatment frequencies calculated from UDD vs. DDDvet have been critically addressed by Kasabova et al. (9) based on treatment data from collectives of broiler chicken, suckling piglet, and fattening pig farms. In the present study, we for the first time applied a similar approach to entire meat production animal sectors in Germany. In particular, for the six animal populations that are subject to the benchmarking system according to the German Medicinal Products Act, (i) we investigated how farm-level UDD-based and DDDvet-based treatment frequencies compare, (ii) we quantified the extent to which farms would shift percentile ranks in the German benchmarking system, (iii) we analyzed how differences between the two types of treatment frequencies can be understood as measurement errors introduced by the use of standardized values instead of actual ones, and (iv) we explored how these differences can be traced back to farm practices at the treatment level with respect to animal weight at the time of treatment and dosage.

## Materials and Methods

### Treatment Frequency

The German Antibiotics Minimization Concept (GAMC) defines the following treatment frequency (TF) to be used for the purpose of quantifying AMU per farm:


$$\begin{array}{l} {\text{TF}}_{\text{GAMC}}\left(f\right)=\frac{1}{{\bar{n}}_{\text{HA}}\left(f\right)}\;\sum_{t=1}^{n_{\text{T}}\left(f\right)}\;\left[n_{\text{TA}}\left(t\right)\times d_{\text{T}}\left(t\right)\times n_{\text{I}}\left(t\right)\right]\\ \;=\frac{1}{{\bar{n}}_{\text{HA}}\left(f\right)}\;\sum_{t=1}^{n_{\text{T}}\left(f\right)}\text{ADT}\left(t\right)\;, \end{array}$$

where ${\bar{n}}_{\text{HA}}\left(f\right)$ is the average number of held animals on farm *f* in the half-year period, *t* is indexing all administered treatments from 1 to *n*_T_(*f*), the number of treatments on the farm in the half-year period, and where *n*_TA_(*t*), *d*_T_(*t*), and *n*_I_(*t*) are the number of treated animals, the duration in days, and the number of active ingredients in treatment *t*, respectively. Note that for long acting VMP's the duration days cover the period of sustained drug activity. The product within the square brackets may also be referred to as the number of animal-days treated, or ADT (for a detailed description of our mathematical notation, see Supplementary Material). Thus, TF_GAMC_ [day] is equivalent to the average number of days treated per animal held. However, because the amount of VMP used in a treatment is also recorded in the database, dose-based AMU metrics can be derived, too.

### Used Daily Dose-Based Metrics

The used daily dose-based treatment frequency, TF_UDD_ [day], is defined as


$$\begin{array}{l} {\text{TF}}_{\text{UDD}}\left(f\right)=\frac{1}{{\bar{n}}_{\text{HA}}\left(f\right)}\;\sum_{t=1}^{n_{\text{T}}\left(f\right)}\;\sum_{i=1}^{n_{\text{I}}\left(t\right)}\frac{m\left(t,i\right)}{{\bar{w}}_{\text{TA}}\left(t\right)\times \text{UDD}\left(t,i\right)}\\ \;=\frac{1}{{\bar{n}}_{\text{HA}}\left(f\right)}\;\sum_{t=1}^{n_{\text{T}}\left(f\right)}\;\sum_{i=1}^{n_{\text{I}}\left(t\right)}\frac{m\left(t,i\right)}{\text{UADD}\left(t,i\right)}\;, \end{array}$$

where *m*(*t, i*) is the amount [in mg] of active ingredient *i* in treatment *t*, *n*_*I*_(*t*) is the number of active ingredients contained in the VMP used in treatment *t*, UDD(*t, i*) is the used daily dose [in mg/kg/day] of active ingredient *i* in treatment *t*, and ${\bar{w}}_{\text{TA}}\left(t\right)$ is the average weight of the treated animals [in kg] in treatment *t*. The product of animal weight and used daily dose is the used animal daily dose, or UADD [in mg/day]. It can be shown that the used daily dose-based treatment frequency is identical to the treatment frequency defined in the German Antibiotics Minimization Concept, i.e., TF_GAMC_= TF_UDD_ (for a detailed derivation of this identity, see Supplementary Material).

### Defined Daily Dose-Based Metrics

Similarly, a defined daily dose-based treatment frequency can be calculated, TF_DDDvet_ [day]:


$$\begin{array}{l} {\text{TF}}_{\text{DDDvet}}\left(f\right)=\frac{1}{{\bar{n}}_{\text{HA}}\left(f\right)}\sum_{t=1}^{n_{\text{T}}\left(f\right)}\;\sum_{i=1}^{n_{\text{I}}\left(t\right)}\frac{m\left(t,i\right)}{{\hat{w}}_{\text{TA}}\times \text{DDDvet}\left(i,p\left(t\right)\right)}\\ \;=\frac{1}{{\bar{n}}_{\text{HA}}\left(f\right)}\;\sum_{t=1}^{n_{\text{T}}\left(f\right)}\;\sum_{i=1}^{n_{\text{I}}\left(t\right)}\frac{m\left(t,i\right)}{\text{DADD}\left(t,i\right)}\;, \end{array}$$

where the actual values for daily dose and animal weight are substituted by standardized values, i.e., by the defined daily dose for animals, DDDvet(*i, p*(*t*)) for active ingredient *i* and pharmaceutical characteristics *p* of treatment *t* (see below), and a standardized animal weight, ŵ_TA_. For the animal weights, we adopted the standardized weights defined by the European Medicines Agency (EMA) for calculating the Population Correction Unit (PCU) when comparing AMU across countries (10). Table 1 includes these standardized weights. Defined daily doses for animals [in mg/kg/day] have been specified by EMA (8) per animal species and active ingredient. In addition, the values may depend on the pharmaceutical characteristics *p* of the VMP used in a treatment *t*. For our purposes, we considered a VMP's pharmaceutical characteristics to be a unique combination of the following factors: (1) administration route (oral, parenteral, premix, other), (2) combination product (yes/no), and (3) long-acting product (yes/no). The defined animal daily dose, DADD [in mg/day], is the product of the PCU animal weight and the DDDvet value.

**Table 1:** Animal weights and weight ratios.

	**Minimum**		**Standardized**		**Maximum**	
**Animal population**	**Weight**	**WR**	**Weight**	**WR**	**Weight**	**WR**
Calves (≤ 8 months)	38 kg	0.271	140 kg	1	250 kg	1.786
Cattle (> 8 months)	200 kg	0.471	425 kg	1	800 kg	1.882
Piglets (≤ 30 kg)	5.5 kg	0.22	25 kg	1	31 kg	1.24
Pigs (> 30 kg)	25 kg	0.385	65 kg	1	120 kg	1.846
Chickens	0.04 kg	0.04	1 kg	1	2.5 kg	2.5
Turkeys	0.06 kg	0.009	6.5 kg	1	21.5 kg	3.308

*Minimum and maximum weights according to Kuratorium für Technik und Bauwesen in der Landwirtschaft e.V. (KTBL) (11), standardized weights as defined by EMA for PCU calculation (12). Respective weight ratio (WR) values were calculated as Minimum WR**=**(Minimum weight)/(standardized weight), WR = 1 for standardized weights, and Maximum WR**=**(Maximum weight)/(standardized weight)*.

### Percentile Rank Shift

The hypothetical effect of switching from TF_UDD_ to TF_DDDvet_ in the benchmarking system established by the German Antibiotics Minimization Concept can be investigated by calculating the percentile rank shift, *PRS*, that farms would undergo:


$$\begin{array}{l} \text{PRS}\left(f\right)=100\times \left({\text{PR}}_{\text{DDDvet}}\left(f\right)-{\text{PR}}_{\text{UDD}}\left(f\right)\right). \end{array}$$

Thus, the percentile rank shift is simply the difference between the percentile rank of farm *f* in a TF_DDDvet_-based ranking, PR_DDDvet_(*f*), and the percentile rank of the same farm in a TF_UDDD_-based ranking, PR_UDD_(*f*). Because the German benchmarking takes place twice per year, these shifts in percentile ranking must be evaluated for each half-year period separately. Example, in a half-year period a farm may be ranked in the 90^th^ percentile with respect to TF_UDD_ but in the 80^th^ percentile with respect to TF_DDDvet_ which would yield a percentile rank shift of −10.

In principle, percentile rank shifts can range from −100– +100. Distributions of percentile rank shifts within an animal population are by definition centered at 0 (overall, exactly the same number of farms must shift to lower or higher ranks) but wider distributions indicate more frequent shifts overall. The median absolute percentile rank shift can be used as a measure of the width of the distribution. Example, if the median absolute percentile rank shift is 10 then half of the farms would shift more than +10 percentile ranks up or more than −10 percentile ranks down in an alternative TF_DDDvet_-based benchmarking process.

### Animal Daily Dose Ratio

Because in the German reporting system the weight of the treated animals is not recorded, a direct comparison of the weight at the time of treatment to the standardized weight is not possible. Nor can the UDD be calculated and compared to the DDDvet. However, a comparison of the products of weight and daily dose is feasible, i.e., of used animal daily dose, UADD, and defined animal daily dose, DADD. To that end, we define the animal daily dose ratio, ADDR:


$$\begin{array}{l} \text{ADDR}\left(t,i\right)=\frac{\text{UADD}\left(t,i\right)}{\text{DADD}\left(t,i\right)}=\frac{{\bar{w}}_{\text{TA}}\left(t\right)}{{\hat{w}}_{\text{TA}}}\times \frac{\text{UDD}\left(t,i\right)}{\text{DDDvet}\left(i,p\left(t\right)\right)}\\ \;=\text{DR}\times \text{WR}. \end{array}$$

Accordingly, the ADDR is the product of a dose ratio, DR, and a weight ratio, WR. ADDR = 1 implies that the UADD was equal to the DADD and thus that the weight of the animals at the time of treatment was equal to the standardized weight *and* that the UDD was equal to the DDDvet, or that deviations of the animal weight and the daily dose from their respective standardized values canceled out. ADDR <1 indicates either a treatment of animals of lower weight than the standardized weight or a low UDD compared to the appropriate DDDvet. Conversely, ADDR >1 implies either that animals of higher weight than the standardized weight were treated or that the UDD was high compared to the appropriate DDDvet.

With tabulated minimum and maximum weights for each animal population it is possible to derive ranges covered by the weight ratio, $\text{WR}=\frac{{\bar{w}}_{\text{TA}}}{{ŵ}_{\text{TA}}}$, as shown in Table 1. For the animal populations subject to the German benchmarking system these ranges are wider for poultry, with animals being included from the time of hatching, than for cattle and pigs where animals are included only from the time of weaning and furthermore subdivisions by age or weight have been defined. If for a specific treatment the ADDR were outside of the weight ratio range, this would imply that the UDD must have deviated significantly from the DDDvet.

In order to characterize treatment practices at the farm level we use a weighted average of the ADDR's of all treatments that have been administered on a farm in a given half-year period:


$$\begin{array}{l} \bar{\text{ADDR}}\left(f\right)=\;\overset{n_{T}}{\underset{t=1}{\sum }}\;\overset{n_{I}\left(t\right)}{\underset{i=1}{\sum }}\left[\frac{\text{ADT}\left(t,i\right)}{{\text{ADT}}_{\text{total}}\left(f\right)}\times \text{ADDR}\left(t,i\right)\right]. \end{array}$$

A farm's average animal daily dose ratio, $\bar{\text{ADDR}}$, can thus be thought of as quantifying overall treatment practices on a farm in relation to standardized values where each treatment is weighted by its contribution to the total number of animal-days treated. Farms that tend to treat animals at younger ages and thus lower weights than the standardized weight (i.e., ${\bar{w}}_{\text{TA}}\left(t\right)<{ŵ}_{\text{TA}}$ for most treatments *t*) or use doses lower than DDDvet values (i.e., UDD(*t, i*) < DDDvet(*i, p*(*t*)) for most treatments *t* with active ingredients *i* and pharmaceutical characteristics *p*) will exhibit a ratio smaller than one, $\bar{\text{ADDR}}<1$. Conversely, farms with the opposite tendencies (treatment at older ages/higher weights and higher doses) will have a ratio greater than one, $\bar{\text{ADDR}}>\;1$.

In order to make this more illustrative we may consider a concrete example. Let us assume that in a given half-year period three treatments take place on a farm and that in each of these a VMP is administered that contains a single active ingredient. For the first treatment T_1_, we assume that 500 animals 25% lighter than the standardized weight are treated for 1 day with a dosage 33% less than the DDDvet. For this treatment, the animal daily dose ratio is $\text{ADDR}=\frac{3}{4}\times \frac{2}{3}=0.5$ and the number of animal-days treated is ADT = 500 × 1 = 500. For the second treatment T_2_, we assume that 75 animals of half the standardized weight are treated for 2 days at twice the DDDvet, yielding $\text{ADDR}=\frac{1}{2}\times 2=1$ and ADT = 75 × 2 = 150. Finally, for the third treatment T_3_ we assume 70 animals weighing 33% more than the standardized weight, a treatment duration of 5 days and a dosage 50% higher than the DDDvet, i.e. $\text{ADDR}=\frac{4}{3}\times \frac{3}{2}=2$ and ADT = 70 × 5 = 350. The total number of animal-days treated on this farm is ADT_total_ = 1000, and the average animal daily dose ratio would calculate to $\bar{\text{ADDR}}=\frac{500}{1000}\times 0.5+\frac{150}{1000}\times 1+\frac{350}{1000}\times 2=\;1.1$.

**Figure 1:**
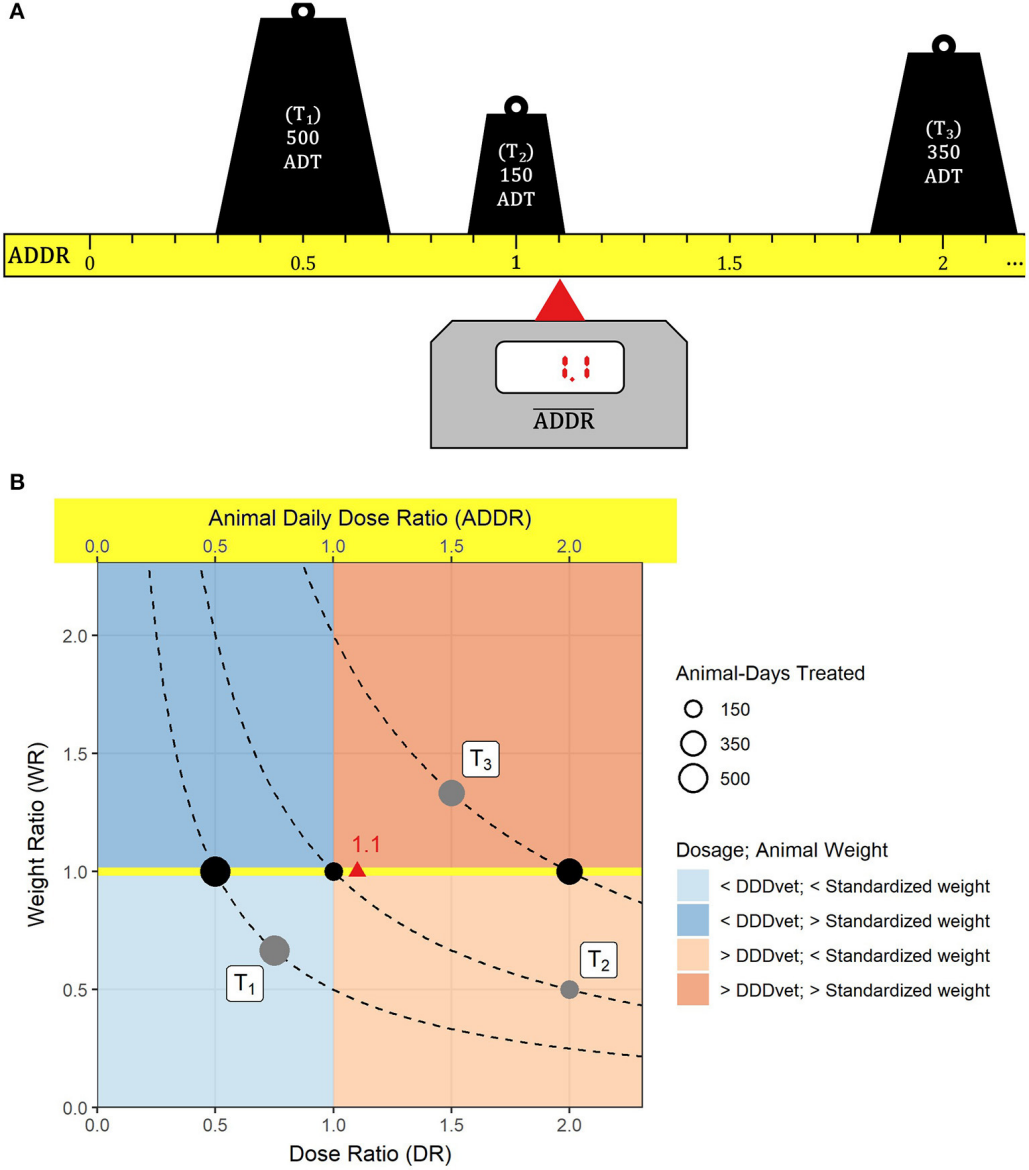
An example illustrating the average animal daily dose ratio. Three treatments take place on a farm: (T_1_) 500 animal-days treated (ADT) with an animal daily dose ratio (ADDR) of 0.5, (T_2_) ADT = 150, ADDR = 1 and (T_3_) ADT = 350, ADDR = 2. **(A)** Each treatment is represented by a weight according to the number of animal-days treated that is located on a ruler measuring the animal daily dose ratio on a linear scale (the ruler is assumed to be weightless and may extend further to the right if necessary). The average animal daily dose ratio ($\bar{\text{ADDR}}=1.1$) marks the point of support on the ruler where the setup balances out. **(B)** The treatments are shown as gray circles on a Cartesian plane where the abscissa of the circle's center is the dose ratio, its ordinate is the weight ratio, and where the area of the circle represents the number of animal-days treated. Each treatment is located on a hyperbole (dashed curves) along which the ADDR is constant. Therefore, in terms of the animal daily dose ratio the black circles are equivalent to the original treatments T_1_, T_2_, and T_3_. They are located on a yellow strip that is equivalent to the ADDR ruler in **(A)** and whose values can be read off the upper abscissa. The red triangle marks the $\bar{\text{ADDR}}$ value.

Figure 1 illustrates the farm's average animal daily dose ratio as the point of support on a scale in equilibrium, using the numbers from the previous example. The ruler in Figure 1A is assumed to be weightless (i.e., it does not affect the balance) but may extend further to the right if the ADDR of a treatment so requires. Figure 1B shows the treatments as circles sized according to their respective animal-days treated on a Cartesian plane with the dose ratio as the abscissa and the weight ratio as the ordinate. The plane can be divided into four regions with respect to standardized dosages and weights. Treatment T_1_ is located in the lower left region where both dosage and animal weight are below their standardized values. Treatment T_2_ is in the lower right region, with a dosage above but an animal weight below the standardized value, and treatment T_3_ is in the upper right region where both dosage and animal weight are higher than the standardized values. In this example, there is no treatment in the upper left region where dosage would be below but animal weight above the standardized value. Because the ADDR is the product of the dose ratio and the weight ratio, all treatments on the same hyperbole are equivalent with respect to the ADDR. Therefore, the black circles in Figure 1B may be used as surrogates for the original treatments. The surrogate treatments are placed on a yellow strip just like the ruler in Figure 1A only viewed from above, and the $\bar{\text{ADDR}}$ can be visualized accordingly as the point of support. The hyperbole that treatment T_2_ is located on also subdivides a farm's treatments with respect to their contribution to its average animal daily dose ratio: treatments left (right) of the hyperbole contribute to an $\bar{\text{ADDR}}$ smaller (greater) than one.

The illustrations in Figure 1 also show that the average animal daily dose ratio has an inherent tendency toward values greater than one because the plane is bounded at the bottom and left and also at the top (compare maximum weight ratios in Table 1) but may extend much further to the right if used daily doses are multiples of DDDvet values.

It is possible to show (for more details, see Supplementary Material) that the average animal daily dose ratio is exactly the inverse of the treatment frequency ratio of the UDD-based to the DDDvet-based treatment frequency, $\text{TFR}\left(f\right)=\frac{\text{T}{\text{F}}_{\text{UDD}}\left(f\right)}{\text{T}{\text{F}}_{\text{DDDvet}}\left(f\right)}$ (defined analogously to the treatment-level ADDR). Because of this inverse relationship, we can use a farm's average animal daily dose ratio to determine the treatment frequency of the farm if standardized values were to be used for animal weights and dosages instead of the actual values:


$$\begin{array}{l} \text{T}{\text{F}}_{\text{DDDvet}}\left(f\right)=\bar{\text{ADDR}}\left(f\right)\times \text{T}{\text{F}}_{\text{UDD}}\left(f\right). \end{array}$$

Example, if a farm predominantly treats very young animals such that $\bar{\text{ADDR}}=0.5$ it would appear that animals on this farm were treated only half as frequently as actually were the case. Conversely, if on a farm treatments were often overdosed such that $\bar{\text{ADDR}}=3$ the DDDvet-based treatment frequency would overestimate the actual frequency at which animals were being treated by a factor of three. This shows that farms will be differently affected by the use of standardized values depending on the farms' treatment practices.

### Estimation Errors

The use of standardized weights and defined daily doses for animals instead of the true values, i.e., actual treatment weights and used daily doses, introduces estimation errors at the treatment and consequently at the farm level. Because the values of both the UDD-based and DDDvet-based metrics span multiple orders of magnitude, it is expedient to use logarithms when calculating estimation errors.

At the treatment/active ingredient level, we define:


$$\begin{array}{l} \text{Error}\left(t,i\right)=\log_{10}\left(\text{DADD}\left(t,i\right)\right)-\log_{10}\left(\text{UADD}\left(t,i\right)\right)\\ \;=\begin{array}{c} \log_{10}\left(\text{ADDR}\left(t,i\right)\right) \end{array}. \end{array}$$

Analogously, at the farm level, we define the error as:


$$\begin{array}{l} \text{Error}\left(f\right)=\log_{10}\left({\text{TF}}_{\text{DDDvet}}\left(f\right)\right)-\log_{10}\left({\text{TF}}_{\text{UDD}}\left(f\right)\right)\\ \;=\begin{array}{c} \log_{10}\left(\bar{\text{ADDR}}\left(f\right)\right) \end{array}. \end{array}$$

The distributions of the animal daily dose ratio and the *average* animal daily dose ratio may therefore be interpreted as reflecting population wide treatment practices with respect to standardized animal weights and daily doses as well as representing error distributions when using standardized values instead of actual ones.

For the comparison of entire animal populations with regard to the introduced estimation error, we use the mean squared error, MSE, of the farm-level errors:


$$\begin{array}{l} \text{MSE}=\frac{1}{\text{N}}\overset{\text{N}}{\underset{f=1}{\sum }}{\left[\begin{array}{c} \log_{10}\left(\bar{\text{ADDR}}\left(f\right)\right) \end{array}\right]}^{2}. \end{array}$$

Here, N is the number of farms in the animal population considered.

### Study Data

The AMU data used in this study covers the seven half-year periods from the second semester of 2014 to the second semester of 2017. The same data were used in the evaluation of the German Antibiotics Minimization Concept (13), but for the present study we applied some slight modifications. Because we aimed to compare UDD-based and DDDvet-based treatment frequencies, we only included treatments for which explicit DDDvet values were available from EMA (8) in order to avoid bias deriving from imputation of missing values. This also applies to long acting VMP's. If DDDvet values specific to long acting injectable products were assigned by EMA, these were used. Otherwise, DDDvet values for conventional injectables were used. Because EMA has yet to publish DDDvet values for long acting injectable VMP's containing gamithromycin, tildipirosin or tulathromycin, all treatments with such products were excluded for our analyses. For each of the half-year periods we excluded farms without antimicrobial treatments in that period. Both types of treatment frequencies are zero in that case, irrespective of whether they are based on *used* or *defined* daily doses.

Furthermore, for the present study we implemented a slightly different method of outlier detection. Flor et al. (13) developed checks to identify implausibly high animal weights at the time of treatment by using DDDvet values (imputed if necessary) to estimate animal weights at the time of treatment and comparing those weights to maximum weights for the appropriate animal group as tabulated in Table 1. Then, treatments with estimated weights larger than three times the maximum weight were considered outliers and excluded from further analysis. Note that for the active ingredient colistin, Flor et al. (13) used a threshold of 20 instead of three because treatment practices among German veterinarians seemed to deviate so significantly from DDDvet values that otherwise a large fraction of colistin treatments would have been excluded. For the present study, we adopted the following approach. We calculated the UADD for each treatment and compared it to the appropriate DADD specified by the active ingredient and pharmaceutical characteristics of the VMP used. Then, we excluded treatments if the UADD was larger than 10^2.5^≈316.2278 times the median of all treatments with the same active ingredient and pharmaceutical characteristics or if it was below one 316.2278^th^ of the median value. Note that these threshold constitute the middle between 100 and 1000 and between $\frac{1}{100}$ and $\frac{1}{1000}$ on a logarithmic scale. With this approach we were able to detect and subsequently exclude data entry errors where by choosing the wrong unit (e.g., mg instead of g or vice versa) the UADD was off by a factor of 1,000 or one 1,000^th^. The Supplementary Material include figures that show UADD distributions before and after outlier exclusion.

In addition, for each animal population we only included active ingredients with at least 35 treatments over all seven half-year periods (i.e., on average at least five treatments per half-year period) in order to base the outlier detection on reliable median values. In total, we included 205,894 farms (counting each half-year period that a farm is included separately) and 2,187,583 treatments (where for combination VMP's each active antimicrobial ingredient is counted as a separate treatment).

Data was prepared with the KNIME analytics platform, version 3.7.2 (14), all analyses were performed using the R statistical software, version 3.6.3 (15).

## Results

### Used Daily Dose-Based vs. Defined Daily Dose-Based Treatment Frequency

**Figure 2:**
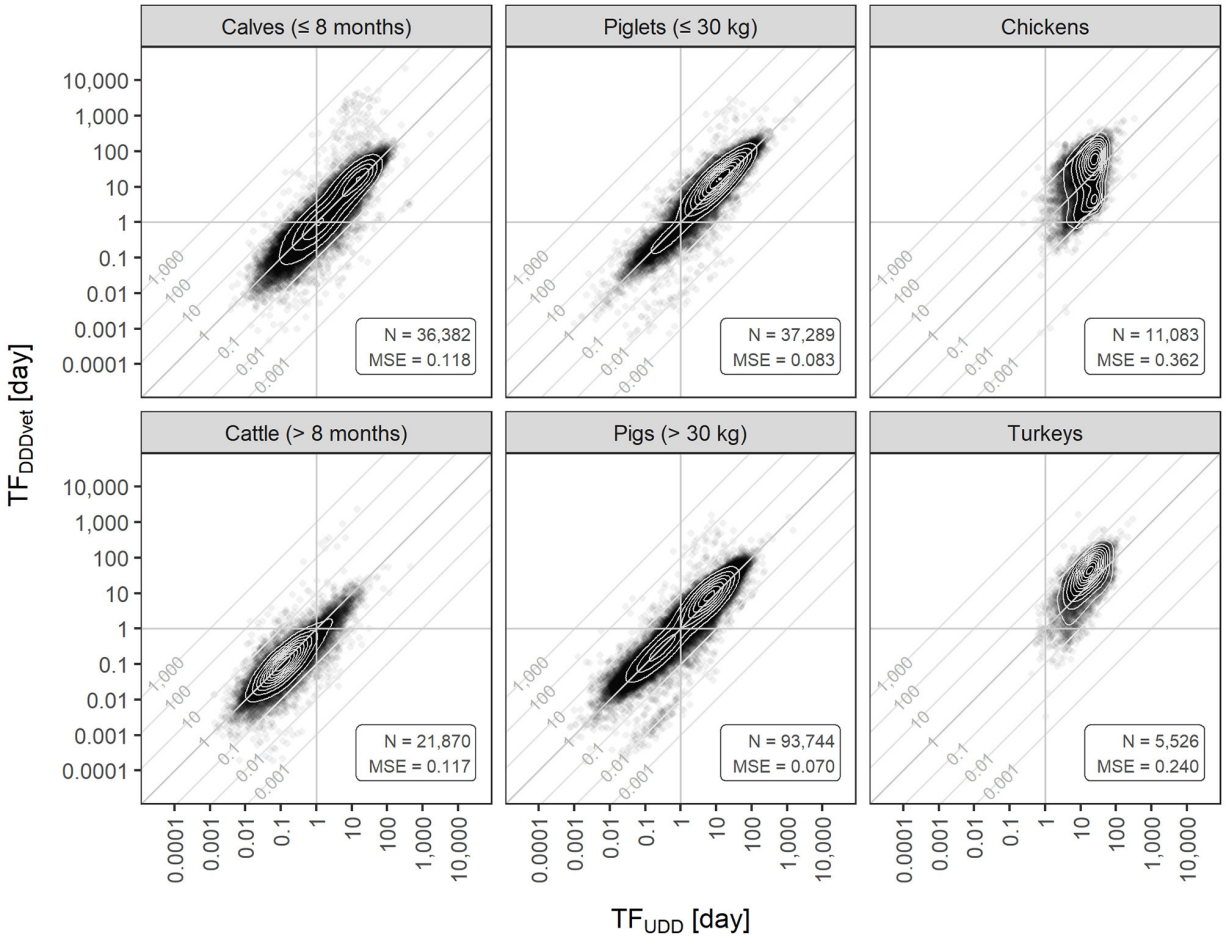
Farm-level DDDvet-based treatment frequencies (TF_DDDvet_) vs. UDD-based treatment frequencies (TF_UDD_) for each of the six animal populations covered by the German benchmarking system. Both axes are log-scaled. Diagonal lines depict isoclines along which the average animal daily dose ratio ($\bar{\text{ADDR}}$) has the same value. Contour lines show the two-dimensional density. Data is from all half-year periods 2014-2 to 2017-2. N, number of farms; MSE, mean squared error with respect to the main diagonal where $\bar{\text{ADDR}}=\;1$.

In the available data, the ranges of both TF_UDD_ and TF_DDDvet_ values covered multiple orders of magnitude, but ranges differed between the two types of treatment frequencies and the six animal populations. In pigs and cattle of both age or weight groups, UDD-based treatment frequencies spanned six orders of magnitude, whereas in poultry they only covered four orders of magnitude. DDDvet-based treatment frequencies spanned at least seven orders of magnitude in all animal populations. This is apparent from Figure 2 where both treatment frequencies are plotted against one another, i.e., each farm is represented by a pair of TF values (TF_UDD_, TF_DDDvet_) per half-year period (note that both axes in the chart are logarithmically scaled). In general, TF_DDDvet_ values followed TF_UDD_ values, i.e., farms clustered along the main diagonal where both treatment frequencies have the same value and the farm-level average animal daily dose ratio is one, $\bar{\text{ADDR}}=1$. However, vertical deviations from the main diagonal also spanned multiple orders of magnitude. Example, farms holding calves (≤ 8 months) with TF_UDD_ = 1 day had designated TF_DDDvet_ values from 0.01 days up to more than 100 days.

The contour lines in the clouds of points indicated bimodal two-dimensional distributions for farms holding calves (≤ 8 months), piglets (≤ 30 kg), pigs (> 30 kg), and chickens suggesting the existence of two subpopulations of farms with different frequencies or practices of treatment, whereas for farms holding cattle (> 8 months) and turkeys only one peak was present. Furthermore, the peaks for calves (≤ 8 months), cattle (> 8 months), piglets (≤ 30 kg), and pigs (> 30 kg) were located right on the main diagonal whereas for chickens and turkeys, they were not. The major peak for chickens as well as the only peak for turkeys were above the main diagonal indicating that for most farms, their TF_DDDvet_ value was larger than their TF_UDD_ value. In farms holding chickens, a second, smaller peak existed below the main diagonal suggesting a subpopulation of farms where this relation between the two types of treatment frequencies was the opposite.

The MSE is a measure of the average vertical deviation from the diagonal (where $\bar{\text{ADDR}}=\;1$) and thus a measure of the average deviation between the two types of treatment frequencies. In our dataset, it was smallest for pigs (> 30 kg) and piglets (≤ 30 kg), and it was highest for turkeys and chickens (for MSE values, see annotations in Figure 2). The MSE was intermediate for calves (≤ 8 months) and cattle (> 8 months). This measure can also be thought of as the mean of the squared $\bar{\text{ADDR}}$ distribution on a logarithmic scale.

For pigs (> 30 kg) and piglets (≤ 30 kg), and to a lesser extent, for calves (≤ 8 months) and cattle (> 8 months), a number of farms clustered along the diagonal along which $\bar{\text{ADDR}}=0.01$, i.e., their AMU as measured by TF_UDD_ was about 100 times their standardized TF_DDDvet_ value. This might reflect a problem with data entry as most of these data points belong to the second semester of 2014, i.e., the first half-year period when the German AMU reporting system was still new.

**Figure 3:**
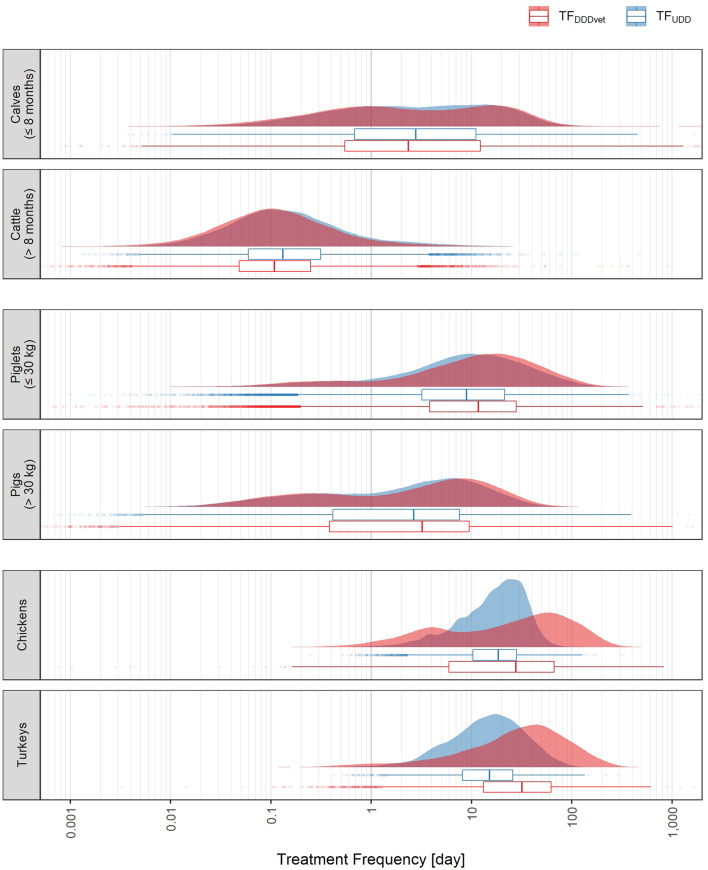
Distributions of UDD-based treatment frequencies, TF_UDD_ (blue), and DDDvet-based treatment frequencies, TF_DDDvet_ (red), for each of the six animal populations covered by the German benchmarking system. The abscissa is log-scaled. Data is from all half-year periods 2014-2 to 2017-2.

The distributions of TF_UDD_ and TF_DDDvet_ are shown in Figure 3. These distributions are equivalent to the marginal distributions of the scatterplots in Figure 2. For farms holding cattle or pigs of both age/weight categories, both distributions showed similar shape and location (note however the logarithmic scale). For calves (≤ 8 months), the median TF_DDDvet_ was 15.5% lower than the median TF_UDD_, and for cattle (> 8 months) it was 17.8% lower, whereas for piglets (≤ 30 kg) and pigs (> 30 kg), it was higher by 31.4% and 21.8%, respectively. For chickens and turkeys, differences in shape and location between the two treatment frequency distributions were much more pronounced. For chickens, the median TF_DDDvet_ was 49.8% higher than the median TF_UDD_, and for turkeys it was more than twice as high (111% higher).

In general, for animal populations that showed treatment frequency distributions with two peaks, this two-peak feature became more pronounced with defined daily dose-based treatment frequencies. Selected quantiles (5%, 25%, 50%, 75%, and 95%) of the two treatment frequency distributions are given in Table 2. Note that the 50% and 75% quantiles cannot be directly compared to AMU indicators published by the German Federal Ministry of Food and Agriculture^1^ because for the present study, farms without AMU were excluded.

**Table 2:** Quantiles of the distributions of **(A)** UDD-based treatment frequencies, TF_UDD_, and **(B)** DDDvet-based treatment frequencies, TF_DDDvet_, for each of the six animal populations covered by the German benchmarking system.

	**5%**	**25%**	**50%**	**75%**	**95%**	**log range**
**(A) UDD-based treatment frequency (TF**_**UDD**_ **[day])**					
Calves (≤ 8 months)	0.12	0.68	2.78	11.08	34.94	2.4
Cattle (> 8 months)	0.02	0.06	0.13	0.31	1.81	2.0
Piglets (≤ 30 kg)	0.26	3.19	8.94	21.44	63.88	2.4
Pigs (> 30 kg)	0.06	0.42	2.66	7.56	21.79	2.6
Chickens	3.55	10.31	18.45	28.25	42.67	1.1
Turkeys	3.16	8.15	15.14	25.88	51.14	1.2
**(B) DDDvet-based treatment frequency (TF**_**DDDvet**_ **[day])**					
Calves (≤ 8 months)	0.09	0.55	2.35	12.26	39.09	2.6
Cattle (> 8 months)	0.01	0.05	0.11	0.25	1.19	1.9
Piglets (≤ 30 kg)	0.21	3.84	11.74	27.96	79.75	2.6
Pigs (> 30 kg)	0.06	0.38	3.23	9.52	26.50	2.7
Chickens	1.43	5.94	27.64	66.48	148.67	2.0
Turkeys	1.74	13.19	31.88	61.96	143.60	1.9

*The width of each distribution is measured as the log difference between the 95% and the 5% quantile, log range = log_10_(q_**95%**_(TF)/q_**5%**_(TF))*.

It could generally be observed that the DDDvet-based treatment frequency distributions were wider than the UDD-based ones: For all animal populations except cattle (> 8 months) the range between the logarithms of the 95% and 5% quantiles (see column “log range” in Table 2) were wider for the TF_DDDvet_ than for the TF_UDD_ distribution.

### Percentile Rank Shift

**Figure 4:**
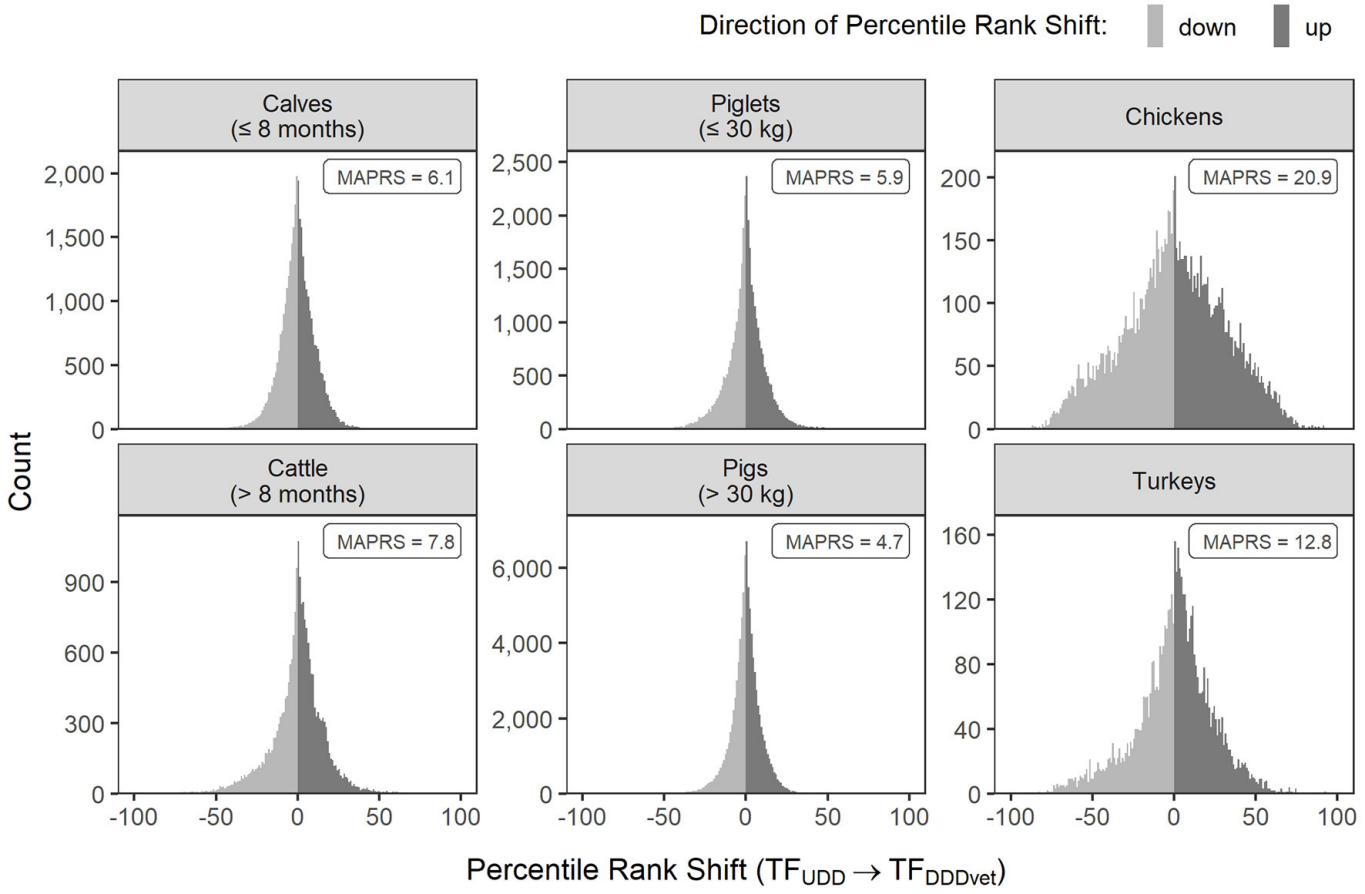
Shift in percentile ranks if farms were ranked by DDDvet-based treatment frequencies (TF_DDDvet_) instead of UDD-based treatment frequencies (TF_UDD_**)** for each of the six animal populations covered by the German benchmarking system. Note that the count axis in each panel is scaled individually. Data is from all half-year periods 2014-2 to 2017-2. MAPRS, median absolute percentile rank shift.

The treatment frequency distributions alone do not determine how farms would fare individually and with respect to other farms if DDDvet-based AMU metrics would be used instead of UDD-based ones. Whether a farm would improve or decline in its percentile rank depends not only on its own average animal daily dose ratio but also on the ratios of the other farms. In order to evaluate the degree of movement of farms up and down the percentile ranks, Figure 4 shows how often percentile rank shifts (in the range from −100 to +100) would have occurred in the six animal populations covered by the German benchmarking system. Because we excluded farms without AMU in the present study, the percentile rank shifts presented here refer to shifts within the subpopulation of farms with AMU^2^.

Percentile rank shifts would occur most frequently for farms holding chickens, followed by farms holding turkeys. Accordingly, the median absolute percentile rank shift would be highest for chickens and turkeys. Half of the farms holding chickens would shift by more than 20.9 percentile ranks, and half of the farms holding turkeys would shift by more than 12.8 percentile ranks. In both of these animal populations, even farms shifting by 50 percentile ranks up or down would not be uncommon.

The distributions were narrower for non-poultry farms, indicating that shifts would be less frequent and smaller. The median absolute percentile rank shift for these animal populations would range between 4.7 [farms holding pigs (> 30 kg)] and 7.8 [farms holding cattle (> 8 months)].

### Animal Daily Dose Ratio

**Figure 5:**
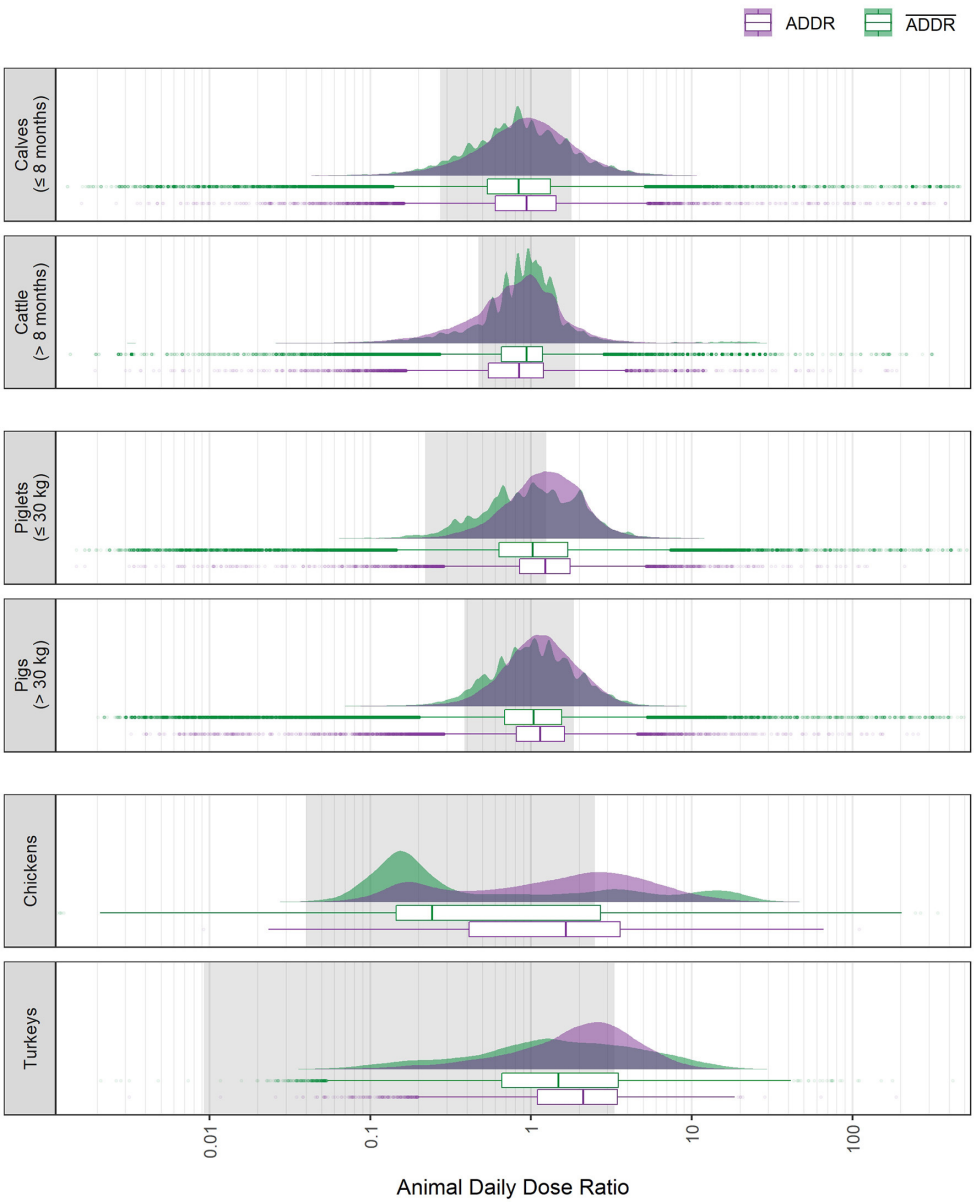
Distribution of the animal daily dose ratio at the individual treatment level (ADDR, purple) and at the farm level ($\bar{\text{ADDR}}$, green) for each of the six animal populations covered by the German benchmarking system. The regions shaded in gray indicate the weight ratio range for each of the animal populations (compare Table 1). The abscissa is log-scaled. Data is from all half-year periods 2014-2 to 2017-2.

For all six animal populations covered by the German benchmarking system the distributions of animal daily dose ratios are shown in Figure 5 on a logarithmic scale, together with the weight ratio ranges as presented in Table 1. Selected quantiles (5%, 25%, 50%, 75%, and 95%) of the treatment-level ADDR and farm-level $\bar{\text{ADDR}}$ distributions are listed in Table 3.

**Table 3:** Quantiles of the distributions of **(A)** treatment-level animal daily dose ratios, ADDR, and **(B)** farm-level average animal daily dose ratios, $\bar{\text{ADDR}}$.

	**5%**	**25%**	**50%**	**75%**	**95%**	**N**	** < Min. WR**	**> Max. WR**
**(A) Treatment level** **(ADDR)**					
Calves (≤ 8 months)	0.235	0.536	0.841	1.323	2.834	598,066	6.2%	13.6%
Cattle (> 8 months)	0.245	0.654	0.941	1.176	2.162	133,237	13.6%	7.1%
Piglets (≤ 30 kg)	0.294	0.632	1.025	1.688	3.030	472,602	2.8%	39.3%
Pigs (> 30 kg)	0.370	0.686	1.037	1.556	2.838	823,983	5.5%	16.6%
Chickens	0.086	0.145	0.243	2.709	15.898	120,162	0.21%	25.9%
Turkeys	0.147	0.657	1.481	3.490	9.467	39,533	0.02%	26.5%
**(B) Farm level** $\left(\bar{\text{ADDR}}\right)$					
Calves (≤ 8 months)	0.255	0.599	0.938	1.431	2.825	36,382	5.7%	15.4%
Cattle (> 8 months)	0.221	0.543	0.845	1.193	2.129	21,870	19.3%	7.4%
Piglets (≤ 30 kg)	0.440	0.848	1.229	1.748	2.896	37,289	1.1%	49.3%
Pigs (> 30 kg)	0.454	0.810	1.141	1.619	2.703	93,744	3.2%	17.9%
Chickens	0.136	0.412	1.648	3.592	8.709	11,083	0.10%	36.9%
Turkeys	0.286	1.099	2.112	3.439	6.492	5,526	0.02%	26.9%

*N, total number of **(A)** treatments (each active ingredient counted separately) or **(B)** farm periods (half-year periods counted separately for each farm). The two right-most columns give percentages of **(A)** treatments with an ADDR or **(B)** farms with an $\bar{\text{ADDR}}$ below the minimum weight ratio, WR, and above the maximum weight ratio*.

At the treatment level, the ADDR distributions reflect sector-wide practices with respect to animal treatment weights and dosages relative to standardized values. The jagged shape of the distributions for cattle and pig populations is due to the fact that for both animal species, VMP's are available that contain active antimicrobial ingredients with very similar DADD values but very different UADD distributions (see Supplementary Figure 2). For poultry this is less often the case which in turn yields smoother ADDR distributions.

The median ADDR for cattle (> 8 months), piglets (≤ 30 kg) and pigs (> 30 kg) was very close to one indicating that on average the standardized values for dosage (DDDvet) and animal weights (PCU) fit well for these animal populations. For calves (≤ 8 months), the median ADDR was below one and for turkeys it was above one. By far the largest deviation from one was observed for chickens with a median ADDR of 0.243. Notably, the ADDR distribution for chickens exhibited three distinct peaks, at approximate ADDR values of 0.15, 3.5, and 15. ADDR distributions for poultry were much wider than those for cattle and pigs, as are weight ratio ranges for chickens and turkeys.

In all of the animal populations, we found treatments with ADDR values outside the weight ratio range. Table 3 lists percentages of treatments that were below the minimum weight ratio or above the maximum weight ratio. Pertaining to 13.6% of treatments, ADDR values below the minimum weight ratio were most common in cattle (> 8 months). In calves (≤ 8 months), piglets (≤ 30 kg) and pigs (> 30 kg), ADDR values below the weight ratio range were present but to a lesser extent. In poultry, such treatments were virtually absent. Treatments with ADDR values above the weight ratio range were generally observed more frequently and in all animal populations. In piglets (≤ 30 kg), nearly 40% of the treatments were located above the maximum weight ratio, and in chickens and turkeys, at least a quarter of the treatments had an ADDR above the weight ratio range.

Figure 5 also shows that at the farm level, the $\bar{\text{ADDR}}$ distributions for cattle and pigs of both age/weight groups appeared as smoother versions of their respective treatment-level ADDR distributions, only slightly shifted to higher values [calves (≤ 8 months), piglets (≤ 30 kg) and pigs (> 30 kg)] or to lower values [cattle (> 8 months)]. For poultry farms, both shape and location of the $\bar{\text{ADDR}}$ distributions differed more strongly from the ADDR distributions. For farms holding chickens, the three-peaked ADDR distribution (with the highest peak at low ADDR values) turned into a two-peaked $\bar{\text{ADDR}}$ distribution, with the higher peak at high $\bar{\text{ADDR}}$ values, at the same time the median moved from *ADDR* = 0.243 to $\bar{\text{ADDR}}=1.648$. For farms holding turkeys, the median $\bar{\text{ADDR}}$ was also considerably larger than the median ADDR.

For all six animal populations, the proportion of farms with $\bar{\text{ADDR}}$ values above the maximum weight ratio was larger than the respective ADDR proportion at the treatment level (see Table 3). Notably, nearly half of the farms holding piglets (≤ 30 kg) and more than a third of farms holding chickens exhibited an $\bar{\text{ADDR}}$ larger than the maximum weight ratio.

## Discussion

In the present study, we compared UDD-based and DDDvet-based AMU metrics using data from whole fattening animal sectors in Germany –calves (≤ 8 months), cattle (> 8 months), piglets (≤ 30 kg), pigs (> 30 kg), chickens, and turkeys. At both the individual treatment and at the farm level, we defined ratios of UDD-based and DDDvet-based metrics. At the treatment level, the animal daily dose ratio (ADDR) is the ratio of the daily dose per animal calculated from UDDs to the daily dose per animal based on DDDvet data. At the farm level, the average animal daily dose ratio ($\bar{\text{ADDR}}$) is a weighted average of the ADDR values of all treatments on a farm in a given half-year period, and the treatment frequency ratio (TFR) is the ratio of the treatment frequency calculated from UDD values to the treatment frequency derived from DDDvet data. We showed that the $\bar{\text{ADDR}}$ is equivalent to the inverse of the TFR and that it can be thought of as an aggregated measure of the treatment practices with respect to standardized values for dosages and animal weights on the farm.

There are three main reasons that a treatment's ADDR may deviate from one:

(R_1_) Individual VMP's recommended dosages can differ from DDDvet values so that even if the recommended dosage of a VMP is used the ADDR can deviate from one. DDDvet values are average recommended dosages per SPC across multiple medicinal products.
(R_2_) Under- or overdosing, i.e., the UDD may differ from the recommended dosage of the product per SPC.
(R_3_) The animal weight at the time of treatment may be different from the standardized weight.

Without further information on animal weight or UDD at the treatment level, these effects can not be distinguished, and as a consequence the ADDR distributions and subsequently the $\bar{\text{ADDR}}$ and treatment frequency distributions must be regarded as a composite of these effects.

At both the farm and the treatment level and for all animal populations considered, we found varying degrees of discrepancies between the UDD- and DDDvet-based metrics, manifested in distributions of the $\bar{\text{ADDR}}$ and ADDR spanning multiple orders of magnitude. In all animal populations, a considerable proportion of treatments had ADDR values that could not be fully explained by animal weights at the time of treatment (see reason R_3_ above). Accordingly, this indicates either large discrepancies between recommended dosage and DDDvet (R_1_) or under-/overdosing (R_2_). Pertaining to more than a quarter of the treatments, ADDR values above the maximum weight ratio were most common in piglets (≤ 30 kg), turkeys, and chickens. Treatments with ADDR values below the minimum weight ratio only constituted a significant proportion in cattle (> 8 months).

For piglets (≤ 30 kg), the tabulated maximum weight of 31 kg is (in relative terms) much closer to the standardized weight of 25 kg than in any of the other animal populations (see Table 1). This may to some extent explain the high proportion of the ADDR and $\bar{\text{ADDR}}$ distributions above the animal weight ratio range. Nevertheless, treatments would have needed to very frequently occur close to the end of this production stage and presumably with high dosages.

The ADDR distribution for chickens with a median value of 0.243 and the highest peak at even lower values strongly suggests that treatment of young chicks weighing far less than the standardized weight of 1 kg occurred very frequently. While the elicitation of recommended dosages of all VMP's authorized for use in Germany was outside the scope of the present study, it seems unlikely that recommended dosages are commonly 15 or even 20 times the DDDvet values. Therefore, the two peaks of the ADDR distribution that were well-outside the weight ratio range for chickens indicate widespread and strong overdosing.

The evaluation of the German Antibiotics Mimimization Concept indicated that colistin was commonly overdosed to a high degree in pigs and poultry (13). This overdosing of colistin is also apparent in Supplementary Figure 2 where the colistin box stands out the most for chickens, turkeys, and for pigs of both age groups. While colistin may constitute the most extreme case the data clearly shows that deviations from the standardized value of ADDR = 1 occurred for all antibiotic ingredients and in all animal populations albeit to varying extent.

For two of the animal populations that were investigated in the present study, Kasabova et al. (9) estimated animal weights at the time of treatment by assuming that the UDD was equal to the recommended dosage in the SPC's of every VMP used in their dataset. For chickens they derived a median weight of 0.122 kg, and for pigs (> 30 kg) a median weight of 52.1 kg. However, these estimations must be considered to still contain the effect of under- and overdosing. It is straightforward to convert their estimates to values that are similar to the ADDR of our framework but do not contain the effects of reason R_1_ in the list above (because they used recommended doses instead of DDDvet values). Converting the median weights to ADDR values we get $\frac{0.122\text{ kg}}{1\text{ kg}}=0.122$ for chickens and $\frac{52.1\text{ kg}}{65\text{ kg}}=0.802$ for pigs (> 30 kg). The difference to the median ADDR values in the present study (0.243 for chickens and 1.037 for pigs (> 30 kg), see Table 3) may thus be attributed in part to the effect of DDDvet values being averages of recommended dosages and in part to the study populations. In fact, Kasabova et al. (9) considered five VMP's authorized for oral medication in pigs containing the active ingredient tylosin as an example and found recommended dosages between 4.5 und 25 mg/kg/day whereas the corresponding DDDvet is 12 mg/kg/day. They argued that for pigs, the discrepancies between TF_DDDvet_ and TF_UDD_ they observed in their dataset were caused mainly by such deviations of recommended dosages from DDDvet values. However, while Kasabova et al. (9) analyzed treatment data from 40 chicken and 449 pig holding farms in 2014 (i.e., during two half-year periods), the present study covers treatment data for whole production sectors in Germany during a span of seven half-year periods. In the tylosin example above, factors in the range of one third to two can be explained by deviations of the recommended dose from the DDDvet value. Using this as a baseline (for R_1_ in the numerated list above) and taking into account the weight ratio ranges (for R_3_), our results suggest that overdosing (R_2_) played a significant role at least in piglets (≤ 30 kg), chickens, and turkeys whereas underdosing may indeed not have been of significance even in cattle (> 8 months).

At the farm level, different treatment practices such as choice of VMPs, frequent overdosing or treating animals predominantly at young ages (and thus low body weights) all contribute to the $\bar{\text{ADDR}}$ and thus determine how strongly the calculation of treatment frequencies would be affected if TF_DDDvet_ was used instead of TF_UDD_. In calves (≤ 8 months), cattle (> 8 months), piglets (≤ 30 kg), pigs (> 30 kg), $\bar{\text{ADDR}}$ distributions overall resembled ADDR distributions, suggesting that treatment practices at the farm level generally reflected practices at the treatment level across the whole production sector. Furthermore, $\bar{\text{ADDR}}$ distributions were fairly symmetrical with median values close to one. Consequently, the distributions of the two types of treatment frequencies showed similar shapes. Nevertheless, median TF_DDDvet_ values ranged from 17.8% lower [in cattle (> 8 months)] to 31.4% higher [in piglets (≤ 30 kg)] than their TF_UDD_ counterparts, revealing considerable errors in AMU measurement.

In chickens and turkeys, $\bar{\text{ADDR}}$ distributions at the farm level differed substantially from ADDR distributions at the treatment level. For chickens the median $\bar{\text{ADDR}}$ was 1.648, i.e., much higher than the median ADDR of 0.243. This increase may be understood by considering the balanced ADDR scale presented in Figure 1: Even if on a farm nine out of ten treatments occur in young chicks –say with a small ADDR of 0.1–, a single treatment that is heavily overdosed –say with an ADDR of 20– will shift the balance toward an $\bar{\text{ADDR}}$ of 2.09 (assuming that all 10 treatments comprise the same number of animal-days treated). Similar effects presumably yielded a median $\bar{\text{ADDR}}$ in turkeys of 2.112 (compared to a median ADDR of 1.481). Correspondingly, median treatment frequencies in poultry were affected more strongly than in cattle and pigs. In chickens, the median TF_DDDvet_ was 49.8% higher than the median TF_UDD_, and in turkeys, it was more than twice as high. I.e., errors in AMU measurement would exceed those in cattle and pigs.

In the present study, we considered treatment frequencies as AMU indicators. From this perspective, the UDD-based treatment frequency can be considered to indicate true AMU on a farm and thus be used as a “gold standard” against which the performance of the DDDvet-based treatment frequency can be investigated. However, with respect to the development of antibiotic resistance also other AMU measures such as the quantity of antimicrobials and their metabolites excreted by treated animals into the environment may be of concern, so our use of the term “error” should be understood only with respect to estimating treatment frequencies and not with respect to estimating AMU *per se*. For consideration of the overall impact of AMU on animal health, environmental health and public health, looking from different angels on AMU may be valuable.

In benchmarking systems constructed to target and reprimand high user farms, AMU measurement errors are problematic in and of themselves. However, if measurement errors are not randomly distributed among farms but instead associated with treatment practices then problems become even more severe. Our results for percentile rank shifts show that benchmarking would be much more errorprone if standardized dosages and animal weights were used, especially in poultry farms. While in the cattle and pig sectors farms would on average shift between 4.7 and 7.8 percentile ranks with DDDvet-based compared to UDD-based metrics, farms holding turkeys would on average shift 12.8 percentile ranks, and farms holding chickens even 20.9 percentile ranks. These numbers do not take into account farms without AMU, so shifts would be smaller in practice. However, differences between the animal populations would be even more pronounced because the proportion of farms without AMU is larger in the pig and cattle sector than in the poultry sector (13).

The fact that farms holding chickens would undergo the most frequent and largest percentile rank shifts can be ascribed to the existence of two distinct types of treatment practices apparent in the two-peaked $\bar{\text{ADDR}}$ distribution. According to the $\bar{\text{ADDR}}$ taking a low or high value in the two peaks, we may call the two types L and H. Figure 2 indicates that the true (UDD-based) treatment frequency for both types is similar. The L-type features farms which presumably only ever treated young chicks yielding an underestimation of their treatment frequency in a DDDvet-based system. On the other hand, H-type farms appear to at least occasionally have administered highly overdosed treatments resulting in an overestimated treatment frequency. During a benchmarking process, L-type farms would thus be rewarded and H-type farms would be penalized.

It is worth noting that our main conclusions are independent from the exact value that is used as the standardized animal weight at the time of treatment. All the DADD and TF_DDDvet_ values would change, and one would have to take this into account when interpreting a farm's average animal daily dose ratio. However, all farms would be affected in the same way yielding the exact same shifts in the percentile rankings as with the standardized weight used in the present study.

Several countries have developed national DDDvet versions taking into account only VMP's authorized for use in the respective country, addressing to some extent the estimation errors originating from R_1_ above and potentially making it more reliable to detect common overdosing behavior as described above for colistin. For example, Bosman et al. (16) assigned Canadian defined daily doses for animals (DDDvetCA) based on antimicrobial products authorized for use in Canada using EMA's principles on assigning DDDvet values (17). Echtermann et al. (18) established Swiss defined daily doses for animals (DDDch) and found that in Switzerland daily doses tended to be lower than the corresponding EMA doses. In Germany, however, because the German Antibiotics Minimization Concept is built on UDD-based treatment frequencies, national DDDvet values have never been assigned. Furthermore, even with nationally defined daily doses for animals, individual VMP's recommended dosages will still differ from such values.

As we have shown, whether AMU is measured by UDD- or DDDvet-based treatment frequencies may affect if a farm is considered a high user. Generally in any benchmarking system, the choice of AMU metric will affect the identification of high usage farms. Example, for Swiss pig farms Kuemmerlen et al. (19) investigated how well two metrics, the number of DDDch's and the number of treatments, both taken per animal and year, agreed when benchmarking levels of antimicrobial usage and identifying high users. They found that although the two metrics were strongly correlated and showed broad agreement certain proportions of farms were categorized differently, and that these proportions varied by age category and benchmarking threshold.

While we focused on the numerator of treatment frequencies as the unit of AMU measurement, the denominator, i.e., the animal population at risk of antimicrobial treatment, may be subject to its own measurement errors. In the German benchmarking system, the animal population at risk is defined as the average number of animals held on a farm in a half-year period. This number is calculated from animal movements (entries and exits) accurate to the day. For plausibility checks of animal movements prior to calculation of average animal numbers, see Flor et al. (13). Just as with imprecise numerators, imprecise denominators can result in farms being wrongly ranked, too. However, these problems do not factor into any of the analyses presented in this study as the denominator is not used in animal daily dose ratios and cancels out during calculation of treatment frequency ratios.

The analyses presented here are based on the actual data collected in the GAMC. Conditions in other countries or animal populations not targeted in the system will certainly vary in detail. However, the populations covered are the major meat production sectors in Germany as well as most other countries and the fundamental issues described are expected also to be valid in other populations and other countries.

## Conclusion

Treatment frequencies based on used daily doses take into account actual weights of animals at the time of treatment and actual dosages. Using pre-defined standardized animal weights and defined daily doses for animals as surrogates requires less extensive data recording. However, it also introduces errors into the calculation of antimicrobial use metrics which aim to estimate the number of treatments or treated animals at farm level. We defined an “average animal daily dose ratio” that is an aggregate measure of these errors at the farm level. It can also be used to quantify a farm's treatment practices relative to standardized animal weights and dosages.

We showed that in all animal populations for which AMU is regulated by the German benchmarking system, animals were frequently treated with animal daily doses outside of plausible animal weight ranges, albeit to varying degrees. This could be an effect of deliberate under- or overdosing. It could also indicate that recommended dosages per SPC deviate from defined daily doses for animals. To assess the relative importance of these two effects, more detailed information at the treatment level is necessary than is currently available in the German AMU monitoring system.

In benchmarking systems that employ treatment frequencies to rank farms according to their antimicrobial use and reprimand high users, the use of standardized values would frequently shift farms in the rankings and in consequence lead to a subset of high users not being targeted and a subset of low users erroneously being targeted. Therefore, used daily dose-based metrics should be preferred in such systems.

## Data Availability Statement

Publicly available datasets were analyzed in this study. This data can be found in aggregated form at https://www.bmel.de/SharedDocs/Downloads/DE/_Tiere/Tierge sundheit/Tierarzneimittel/16-AMG-Novelle-Anlage2.pdf.

## Author Contributions

MF: data curation, formal analysis, methodology, software, validation, visualization, and writing—original draft preparation. MF, B-AT, and AK: conceptualization and writing—review and editing. B-AT and AK: supervision and funding acquisition. All authors contributed to the article and approved the submitted version.

## Conflict of Interest

The authors declare that the research was conducted in the absence of any commercial or financial relationships that could be construed as a potential conflict of interest.

## Publisher's Note

All claims expressed in this article are solely those of the authors and do not necessarily represent those of their affiliated organizations, or those of the publisher, the editors and the reviewers. Any product that may be evaluated in this article, or claim that may be made by its manufacturer, is not guaranteed or endorsed by the publisher.

## Footnotes

^1^ https://www.bmel.de/DE/themen/tiere/tierarzneimittel/entwicklung-kennzahlen-therapiehaeufigkeit.html

^2^These percentile rank shifts can however easily be converted into percentile rank shifts for the whole animal population if the fraction of farms without AMU is known [numbers can be found in Flor et al. (13)]: *p*_0_ = (1−π_0_) × *p*, where π_0_ is the fraction of farms without AMU, *p* is a percentile rank shift as used in this study, and *p*_0_ is the corresponding percentile rank shift if farms without AMU are included.

## Abbreviations

ADDR: Animal Daily Dose Ratio
ADT: Number of Animal-Days Treated
AMU: Antimicrobial Use
DADD: Defined Animal Daily Dose
DDDvet: Defined Daily Dose for animals
EMA: European Medicines Agency
GAMC: German Antibiotics Minimization Concept
MSE: Mean Squared Error
PCU: Population Correction Unit
SPC: Summary of Product Characteristics
TF: Treatment Frequency
TFR: Treatment Frequency Ratio
UADD: Used Animal Daily Dose
UDD: Used Daily Dose
VMP: Veterinary Medicinal Product.
