# Supplementary material for: Contrasting Treatment- and Farm-Level Metrics of Antimicrobial Use Based on Used Daily Dose vs. Defined Daily Dose for the German Antibiotics Minimization Concept
Source: Front Vet Sci. 2022 Jun 27;9:913197. doi: 10.3389/fvets.2022.913197 (PMC9271936; doi:10.3389/fvets.2022.913197)
Supplement: Supplementary file 1 [file Presentation_1.PDF]

Supplementary Material

1 Mathematical notation and derivation of formulas

1.1 Treatment characteristics and other parameters

We characterize each treatment t by the following parameters:

$p(t)$ – pharmaceutical characteristics of the veterinary medicinal product (VMP) administered in treatment t: We define pharmaceutical characteristics a as a unique combination of the following factors: (1) administration route (oral, parenteral, premix, other), (2) combination product (yes/no), and (3) long-acting product (yes/no) emulating the way that DDDvet values are defined by the European Medicines Agency (1):

$\mathrm{DDDvet}(i, p(t))$ – Defined Daily Dose for animals [mg/kg/day] for active ingredient i when administered with characteristics $p(t)$

$I(t)$ – list of active ingredients in the VMP administered in treatment t

$n_{\mathrm{I}}(t) = \mathrm{length}(I(t))$ – number of active ingredients in the VMP administered in treatment t; may be greater than one if combination medicinal products are administered

$m(t, i)$ – for each active ingedient $i \in I(t)$ in treatment t: amount [mg] of active ingredient

$\bar{w}_{\mathrm{TA}}(t)$ – average weight [kg] of treated animals in treatment t; this assumes that all animals treated in the same treatment are of equal or very similar age and thus weight

$n_{\mathrm{TA}}(t)$ – number of treated animals in treatment t

$d_{\mathrm{T}}(t)$ – duration [day] of treatment t; number of days the administered ingredient is antibiotically active

In addition, we define

d_{P} – duration [day] of a half-year period

$\bar{n}_{\mathrm{HA}}(f)$ – average number of housed animals belonging to a specific animal population (such as fattening pigs over 30 kg) on farm f in a half-year period

$n_{\mathrm{T}}(f)$ – number of treatments within a specific animal population (such as fattening pigs over 30 kg) on farm f in a half-year period

$\hat{w}_{\mathrm{TA}}$ – standard weight [kg] of treated animals of a specific animal population (such as fattening pigs over 30 kg), e.g. the weight used in the calculation of population correction units (PCU)

1.2 Used Daily Dose-based Treatment Frequency

The used daily dose, UDD [mg/kg/day], of active ingredient i in treatment t is

$$\mathrm{UDD}(t,i) = \frac{m(t,i)}{n_{\mathrm{TA}}(t) \times \bar{w}_{\mathrm{TA}}(t) \times d_{\mathrm{T}}(t)}.$$

The product of animal weight at the time of treatment and the used daily dose is the used animal daily dose, UADD [mg/day]:

$$\begin{aligned}\mathrm{UADD}(t,i) &= \bar{w}_{\mathrm{TA}}(t) \times \mathrm{UDD}(t,i)\\ &= \frac{m(t,i)}{n_{\mathrm{TA}}(t) \times d_{\mathrm{T}}(t)}.\end{aligned}$$

Hence, the number of animal-days treated, ADT [day], with active ingredient i in treatment t can be expressed in terms of used animal daily doses if the amount of active ingedient is known:

$$\begin{aligned}\mathrm{ADT}(t,i) &= n_{\mathrm{TA}}(t) \times d_{\mathrm{T}}(t)\\ &= \frac{m(t,i)}{\bar{w}_{\mathrm{TA}}(t) \times \mathrm{UDD}(t,i)}\\ &= \frac{m(t,i)}{\mathrm{UADD}(t,i)}.\end{aligned}$$

The total number of animal-days treated in treatment t can be calculated by summation over all active ingredients administered in that treatment:

$$\begin{aligned}\mathrm{ADT}(t) &= \sum_{i=1}^{n_{\mathrm{I}}(t)} \mathrm{ADT}(t,i)\\ &= n_{\mathrm{TA}}(t) \times d_{\mathrm{T}}(t) \times n_{\mathrm{I}}(t).\end{aligned}$$

Similarly, the total number of animal-days treated with active ingredient i on a farm is derived by summation over all treatments in which that ingredient was included:

$$\mathrm{ADT}(i) = \sum_{t \mid i \in I(t)} \mathrm{ADT}(t,i)$$

Restricting the summation to treatments administered with specific pharmaceutical characteristics yields the total number of animal-days treated with active ingredient i administered with characteristics p:

$$\mathrm{ADT}(i,p) = \sum_{t \mid i \in I(t)\ \&\ p=p(t)} \mathrm{ADT}(t,i).$$

Finally, the total number of animal-days treated on farm f is given by

$$\mathrm{ADT}_{\mathrm{total}}(f) = \sum_{t=1}^{n_{\mathrm{T}}(f)} \mathrm{ADT}(t)$$

$$= \sum_{t=1}^{n_T(f)} [n_{TA}(t) \times d_T(t) \times n_I(t)]$$
$$= \sum_{t=1}^{n_T(f)} \sum_{i=1}^{n_I(t)} \frac{m(t,i)}{\mathrm{UADD}(t,i)}.$$

The German Antibiotics Minimisation Concept (GAMC) is build around a farm-level AMU benchmarking process and defines the treatment frequency as the total number of animal-days treated divided by the average number of animals housed on the farm:

$$\mathrm{TF_{GAMC}}(f) = \frac{1}{\bar{n}_{HA}(f)} \sum_{t=1}^{n_T(f)} [n_{TA}(t) \times d_T(t) \times n_I(t)].$$

It is calculated per animal population and twice per year. It takes into account all treatments that were administered on a farm within the respective animal population and in the half-year period under consideration. Thus, the treatment frequency measures the number of days an average animal has been treated in a half-year period.

This treatment frequency can be re-formulated:

$$\mathrm{TF_{GAMC}}(f) = \frac{1}{\bar{n}_{HA}(f)} \sum_{t=1}^{n_T(f)} \mathrm{ADT}(t)$$
$$= \frac{1}{\bar{n}_{HA}(f)} \sum_{t=1}^{n_T(f)} \sum_{i=1}^{n_I(t)} \frac{m(t,i)}{\mathrm{UADD}(t,i)}$$
$$= \frac{1}{\bar{n}_{HA}(f)} \sum_{t=1}^{n_T(f)} \sum_{i=1}^{n_I(t)} \frac{m(t,i)}{\bar{w}_{TA}(t) \times \mathrm{UDD}(t,i)}$$
$$\equiv \mathrm{TF_{UDD}}(f).$$

This shows that the treatment frequency used in the German Antibiotics Minimisation Concept is equivalent to a treatment frequency based on the amounts of active ingredients, the used daily doses, and the average weights of the treated animals at the time of each treatment. We call this a used daily dose-based treatment frequency, $\mathrm{TF_{UDD}}$ [day].

1.3 Defined Daily Dose-based Treatment Frequency

By replacing –for all treatments t– the average weight of the treated animals with a standard animal weight, i.e. $\bar{w}_{TA}(t) \rightarrow \hat{w}_{TA}$, and the *used* daily dose with the appropriate *defined* daily dose for animals, i.e. $\mathrm{UDD}(t,i) \rightarrow \mathrm{DDDvet}(i, p(t))$, we get a defined daily dose-based treatment frequency, $\mathrm{TF_{DDDvet}}$ [day],

$$\mathrm{TF_{DDDvet}}(f) = \frac{1}{\bar{n}_{HA}(f)} \sum_{t=1}^{n_T(f)} \sum_{i=1}^{n_I(t)} \frac{m(t,i)}{\hat{w}_{TA} \times \mathrm{DDDvet}(i, p(t))}$$

$$= \frac{1}{\bar{n}_{\mathrm{HA}}(f)} \sum_{t=1}^{n_{\mathrm{T}}(f)} \sum_{i=1}^{n_{\mathrm{I}}(t)} \frac{m(t,i)}{\mathrm{DADD}(t,i)},$$

where we call $\mathrm{DADD}(t,i) = \widehat{w}_{\mathrm{TA}} \times \mathrm{DDDvet}(i, p(t))$ the defined animal daily dose, DADD [mg/day].

1.4 Animal Daily Dose Ratio

We can define the (unit-less) animal daily dose ratio, ADDR, as the ratio of the *used* to the *defined* animal daily dose of ingredient i in treatment t:

$$\begin{aligned} \mathrm{ADDR}(t,i) &= \frac{\mathrm{UADD}(t,i)}{\mathrm{DADD}(t,i)} \\ &= \frac{\bar{w}_{\mathrm{TA}}(t) \times \mathrm{UDD}(t,i)}{\widehat{w}_{\mathrm{TA}} \times \mathrm{DDDvet}\big(i, p(t)\big)} \\ &= \mathrm{WR}(t) \times \mathrm{DR}(t,i)\,, \end{aligned}$$

where $\mathrm{WR}(t) = \frac{\bar{w}_{\mathrm{TA}}(t)}{\widehat{w}_{\mathrm{TA}}}$ is the weight ratio of the average animal weight at the time of treatment t to the PCU standard weight, and where $\mathrm{DR}(t,i) = \frac{\mathrm{UDD}(t,i)}{\mathrm{DDDvet}(i,p(t))}$ is the dose ratio of the used daily dose of ingredient i in treatment t to the defined daily dose for animals for that ingredient administered with the pharmaceutical characteristics of treatment t. The animal daily dose ratio, therefore, can be understood as the product of two other ratios, where the first ratio, WR, describes how much the actual animal weight at the time of a treatment deviates from the standard PCU weight, and where the second ratio, DR, quantifies how much the dose actually used in a treatment differs from the dose defined by the European Medicines Agency (1). In absence of knowledge about the animal weight at the time of treatment or the used daily dose, the animal daily dose ratio allows us to analyze the combined effect of those two deviations from standard values (PCU weight and DDDvet value).

The animal daily dose ratio has the property that $\mathrm{ADDR}(t,i) > 1$ indicates that active ingredient i has been administered in higher dosage than the defined daily dose suggests, and/or that the animals have been treated at higher weights than the assumed average weight at time of treatment for the animal population. Vice versa, a ratio smaller than one, $\mathrm{ADDR}(t,i) < 1$ indicates that the used dosage of ingredient i was lower than the defined daily dose, and/or that the weight of animals at the time of treatment was lower than the assumed average weight for the animal population. The dose and weight deviations can of course point in different directions, and it is not possible to disentangle them without further knowledge about either one.

1.5 Average Animal Daily Dose Ratio and Treatment Frequency Ratio

With respect to animal weight at the time of treatment and dosage, we can quantify a farm's treatment practices by calculating a weighted average of the ADDR's of all treatments on the farm where each treatment is weighted by its contribution to the total number of animal-days treated on the farm:

$$\overline{\mathrm{ADDR}}(f) = \sum_{t=1}^{n_{\mathrm{T}}} \sum_{i=1}^{n_{\mathrm{I}}(t)} \left[\frac{\mathrm{ADT}(t,i)}{\mathrm{ADT}_{\mathrm{total}}(f)} \times \mathrm{ADDR}(t,i)\right].$$

Farms that tend to treat animals at younger ages and thus lower weights than the PCU standard weights (i.e. $\bar{w}_{\mathrm{TA}}(t) < \hat{w}_{\mathrm{TA}}$ for most treatments) and/or use lower doses than defined daily doses suggest (i.e. $\mathrm{UDD}(t,i) < \mathrm{DDDvet}(i,p(t))$ for most treatments and active ingredients) will exhibit a value smaller than one, $\overline{\mathrm{ADDR}} < 1$. On the other hand, farms with the opposite tendencies (treatment at older ages/higher weights and/or higher doses) will show a value greater than one, $\overline{\mathrm{ADDR}} > 1$.

It can be shown that this weighted ADDR average links the two types of treatment frequencies of the previous paragraphs. In fact, it is the inverse of the treatment frequency ratio, TFR, which we define as the ratio of the *used* to the *defined* daily dose-based treatment frequency:

$$\mathrm{TFR}(f) = \frac{\mathrm{TF_{UDD}}(f)}{\mathrm{TF_{DDDvet}}(f)}.$$

The inverse relationship can be seen via the following re-arrangements:

$$\begin{aligned}
\overline{\mathrm{ADDR}}(f) &= \frac{1}{\mathrm{ADT_{total}}(f)} \sum_{t=1}^{n_{\mathrm{T}}(f)} \sum_{i=1}^{n_{\mathrm{I}}(t)} [\mathrm{ADT}(t,i) \times \mathrm{ADDR}(t,i)] \\
&= \frac{\sum_{t=1}^{n_{\mathrm{T}}(f)} \sum_{i=1}^{n_{\mathrm{I}}(t)} [\mathrm{ADT}(t,i) \times \mathrm{ADDR}(t,i)]}{\sum_{t=1}^{n_{\mathrm{T}}(f)} \sum_{i=1}^{n_{\mathrm{I}}(t)} \mathrm{ADT}(t,i)} \\
&= \frac{\sum_{t=1}^{n_{\mathrm{T}}(f)} \sum_{i=1}^{n_{\mathrm{I}}(t)} \left[\frac{m(t,i)}{\mathrm{UADD}(t,i)} \times \frac{\mathrm{UADD}(t,i)}{\mathrm{DADD}(t,i)}\right]}{\sum_{t=1}^{n_{\mathrm{T}}(f)} \sum_{i=1}^{n_{\mathrm{I}}(t)} \frac{m(t,i)}{\mathrm{UADD}(t,i)}} \\
&= \frac{\frac{1}{\bar{n}_{\mathrm{HA}}(f)} \sum_{t=1}^{n_{\mathrm{T}}(f)} \sum_{i=1}^{n_{\mathrm{I}}(t)} \frac{m(t,i)}{\mathrm{DADD}(t,i)}}{\frac{1}{\bar{n}_{\mathrm{HA}}(f)} \sum_{t=1}^{n_{\mathrm{T}}(f)} \sum_{i=1}^{n_{\mathrm{I}}(t)} \frac{m(t,i)}{\mathrm{UADD}(t,i)}} \\
&= \frac{\mathrm{TF_{DDDvet}}(f)}{\mathrm{TF_{UDD}}(f)} \\
&= \frac{1}{\mathrm{TFR}(f)}.
\end{aligned}$$

Therefore, we can write the following equality:

$$\mathrm{TF_{DDDvet}}(f) = \overline{\mathrm{ADDR}}(f) \times \mathrm{TF_{UDD}}(f).$$

1.6 Estimation Errors

The measures $\mathrm{DADD}(t,i)$ and $\mathrm{TF_{DDDvet}}(f)$ may also be thought of as estimating the true values $\mathrm{UADD}(t,i)$ on the treatment level and $\mathrm{TF_{UDD}}(f)$ on the farm level, respectively. From this perspective, the use of standard values for the animal weights at the time of treatment and the administered daily doses introduces errors into the estimation. Because the values span multiple orders of magnitude, it is expedient to use logarithms when calculating estimation errors.

On the treatment/active ingredient level, we get

$$\begin{aligned}\mathrm{Error}(t,i) &= \log_{10}\big(\mathrm{DADD}(t,i)\big) - \log_{10}\big(\mathrm{UADD}(t,i)\big)\\ &= \log_{10}\left(\frac{\mathrm{DADD}(t,i)}{\mathrm{UADD}(t,i)}\right)\\ &= \log_{10}\big(\mathrm{ADDR}(t,i)\big)\,.\end{aligned}$$

Therefore, the logarithm of the animal daily dose ratio of a treatment also constitutes the treatment-level estimation error.

Similarly, on the farm level an analogous equality holds:

$$\begin{aligned}\mathrm{Error}(f) &= \log_{10}\big(\mathrm{TF_{DDDvet}}(f)\big) - \log_{10}\big(\mathrm{TF_{UDD}}(f)\big)\\ &= \log_{10}\left(\frac{\mathrm{TF_{DDDvet}}(f)}{\mathrm{TF_{UDD}}(f)}\right)\\ &= \log_{10}\big(\overline{\mathrm{ADDR}}(f)\big)\,.\end{aligned}$$

I.e., the logarithm of the average animal daily dose can be interpreted as the error in a farm's treatment frequency estimation that stems from using standard values for animal weights and daily doses instead of actual values.

Finally, on an animal sector level with N farms where AMU is estimated by DDDvet-based instead of being measured by UDD-based treatment frequencies, the mean squared error, MSE, is

$$\mathrm{MSE} = \frac{1}{\mathrm{N}}\sum_{f=1}^{\mathrm{N}}\left[\log_{10}\big(\overline{\mathrm{ADDR}}(f)\big)\right]^2 .$$

The mean squared error can be used to compare animal sectors with respect to how large an AMU estimation error is introduced on average by using DDDvet-based treatment frequencies instead of UDD-based treatment frequencies.

$$\overline{\mathrm{ADDR}}(f) = \sum_{t=1}^{n_{\mathrm{T}}}\sum_{i=1}^{n_{\mathrm{I}}(t)}\left[\frac{\mathrm{ADT}(t,i)}{\mathrm{ADT_{total}}(f)} \times \mathrm{ADDR}(t,i)\right].$$

Farms that tend to treat animals at younger ages and thus lower weights than the PCU standard weights (i.e. $\overline{w}_{\mathrm{TA}}(t) < \widehat{w}_{\mathrm{TA}}$ for most treatments) and/or use lower doses than defined daily doses suggest (i.e. $\mathrm{UDD}(t,i) < \mathrm{DDDvet}(i,p(t))$ for most treatments and active ingredients) will exhibit a value smaller than one, $\overline{\mathrm{ADDR}} < 1$. On the other hand, farms with the opposite tendencies (treatment at older ages/higher weights and/or higher doses) will show a value greater than one, $\overline{\mathrm{ADDR}} > 1$.

It can be shown that this weighted ADDR average links the two types of treatment frequencies of the previous paragraphs. In fact, it is the inverse of the treatment frequency ratio, TFR, which we define as the ratio of the *used* to the *defined* daily dose-based treatment frequency:

$$\mathrm{TFR}(f) = \frac{\mathrm{TF_{UDD}}(f)}{\mathrm{TF_{DDDvet}}(f)}\,.$$

The inverse relationship can be seen via the following re-arrangements:

$$\begin{aligned}
\overline{\mathrm{ADDR}}(f) &= \frac{1}{\mathrm{ADT}_{\mathrm{total}}(f)} \sum_{t=1}^{n_{\mathrm{T}}(f)} \sum_{i=1}^{n_{\mathrm{I}}(t)} [\mathrm{ADT}(t,i) \times \mathrm{ADDR}(t,i)] \\
&= \frac{\sum_{t=1}^{n_{\mathrm{T}}(f)} \sum_{i=1}^{n_{\mathrm{I}}(t)} [\mathrm{ADT}(t,i) \times \mathrm{ADDR}(t,i)]}{\sum_{t=1}^{n_{\mathrm{T}}(f)} \sum_{i=1}^{n_{\mathrm{I}}(t)} \mathrm{ADT}(t,i)} \\
&= \frac{\sum_{t=1}^{n_{\mathrm{T}}(f)} \sum_{i=1}^{n_{\mathrm{I}}(t)} \left[\frac{m(t,i)}{\mathrm{UADD}(t,i)} \times \frac{\mathrm{UADD}(t,i)}{\mathrm{DADD}(t,i)}\right]}{\sum_{t=1}^{n_{\mathrm{T}}(f)} \sum_{i=1}^{n_{\mathrm{I}}(t)} \frac{m(t,i)}{\mathrm{UADD}(t,i)}} \\
&= \frac{\frac{1}{\bar{n}_{\mathrm{HA}}(f)} \sum_{t=1}^{n_{\mathrm{T}}(f)} \sum_{i=1}^{n_{\mathrm{I}}(t)} \frac{m(t,i)}{\mathrm{DADD}(t,i)}}{\frac{1}{\bar{n}_{\mathrm{HA}}(f)} \sum_{t=1}^{n_{\mathrm{T}}(f)} \sum_{i=1}^{n_{\mathrm{I}}(t)} \frac{m(t,i)}{\mathrm{UADD}(t,i)}} \\
&= \frac{\mathrm{TF}_{\mathrm{DDDvet}}(f)}{\mathrm{TF}_{\mathrm{UDD}}(f)} \\
&= \frac{1}{\mathrm{TFR}(f)} \,.
\end{aligned}$$

Therefore, we can write the following equality:

$$\mathrm{TF}_{\mathrm{DDDvet}}(f) = \overline{\mathrm{ADDR}}(f) \times \mathrm{TF}_{\mathrm{UDD}}(f)\,.$$

1.7 Estimation Errors

The measures $\mathrm{DADD}(t,i)$ and $\mathrm{TF}_{\mathrm{DDDvet}}(f)$ may also be thought of as estimating the true values $\mathrm{UADD}(t,i)$ on the treatment level and $\mathrm{TF}_{\mathrm{UDD}}(f)$ on the farm level, respectively. From this perspective, the use of standard values for the animal weights at the time of treatment and the administered daily doses introduces errors into the estimation. Because the values span multiple orders of magnitude, it is expedient to use logarithms when calculating estimation errors.

On the treatment/active ingredient level, we get

$$\begin{aligned}
\mathrm{Error}(t,i) &= \log_{10}\big(\mathrm{DADD}(t,i)\big) - \log_{10}\big(\mathrm{UADD}(t,i)\big) \\
&= \log_{10}\left(\frac{\mathrm{DADD}(t,i)}{\mathrm{UADD}(t,i)}\right) \\
&= \log_{10}\big(\mathrm{ADDR}(t,i)\big)\,.
\end{aligned}$$

Therefore, the logarithm of the animal daily dose ratio of a treatment also constitutes the treatment-level estimation error.

Similarly, on the farm level an analogous equality holds:

$$\mathrm{Error}(f) = \log_{10}\big(\mathrm{TF}_{\mathrm{DDDvet}}(f)\big) - \log_{10}\big(\mathrm{TF}_{\mathrm{UDD}}(f)\big)$$

$$= \log_{10}\left(\frac{\mathrm{TF}_{\mathrm{DDDvet}}(f)}{\mathrm{TF}_{\mathrm{UDD}}(f)}\right)$$
$$= \log_{10}\left(\overline{\mathrm{ADDR}}(f)\right)$$

I.e., the logarithm of the average animal daily dose can be interpreted as the error in a farm's treatment frequency estimation that stems from using standard values for animal weights and daily doses instead of actual values.

Finally, on an animal sector level with N farms where AMU is estimated by DDDvet-based instead of being measured by UDD-based treatment frequencies, the mean squared error, MSE, is

$$\mathrm{MSE} = \frac{1}{\mathrm{N}}\sum_{f=1}^{\mathrm{N}}\left[\log_{10}\left(\overline{\mathrm{ADDR}}(f)\right)\right]^2 .$$

The mean squared error can be used to compare animal sectors with respect to how large an AMU estimation error is introduced on average by using DDDvet-based treatment frequencies instead of UDD-based treatment frequencies.

2 Plausibility checks and outlier removal

We implemented plausibility checks for the treatment data based on outlier detection per animal population, active ingredient, and pharmaceutical characteristics as DDDvet values are defined on this level (1). For each animal population, Supplementary Figure 1 shows how UADD values are distributed per active ingredient/pharmaceutical characteristics before removal of outliers, whereas Supplementary Figure 2 shows the distributions after removal.

Treatment data were classified as outliers if their UADD values were either

- larger than 316.2278 times the median value, or
- smaller than one 316.2278th of the median value,

where median value refers to the distribution of UADD values for the active ingredient/pharmaceutical characteristics checked. Note that $10^{0.5} \approx 316.2278$ which constitutes the middle between 100 and 1000 on a logarithmic scale. By using these thresholds, we presumably detect and subsequently exclude data entry errors where by choosing the wrong unit (e.g. mg instead of g or vice versa) the UADD is off by a factor of 1000 or one 1000th.

The plausibility checks in Flor et al. (2) are similar but rely on estimating animal weights at the time of treatment based on DDDvet rather than UDD values (and imputed DDDvet values where necessary). Furthermore, they only excluded treatment data if animal weights were implausibly high but not if they were implausibly low. For our study, we wanted to avoid the biases introduced by these asymmetrical plausibility checks. Compared to Flor et al. (2), we have included more treatment data with high used daily doses but excluded some data with low used daily doses.

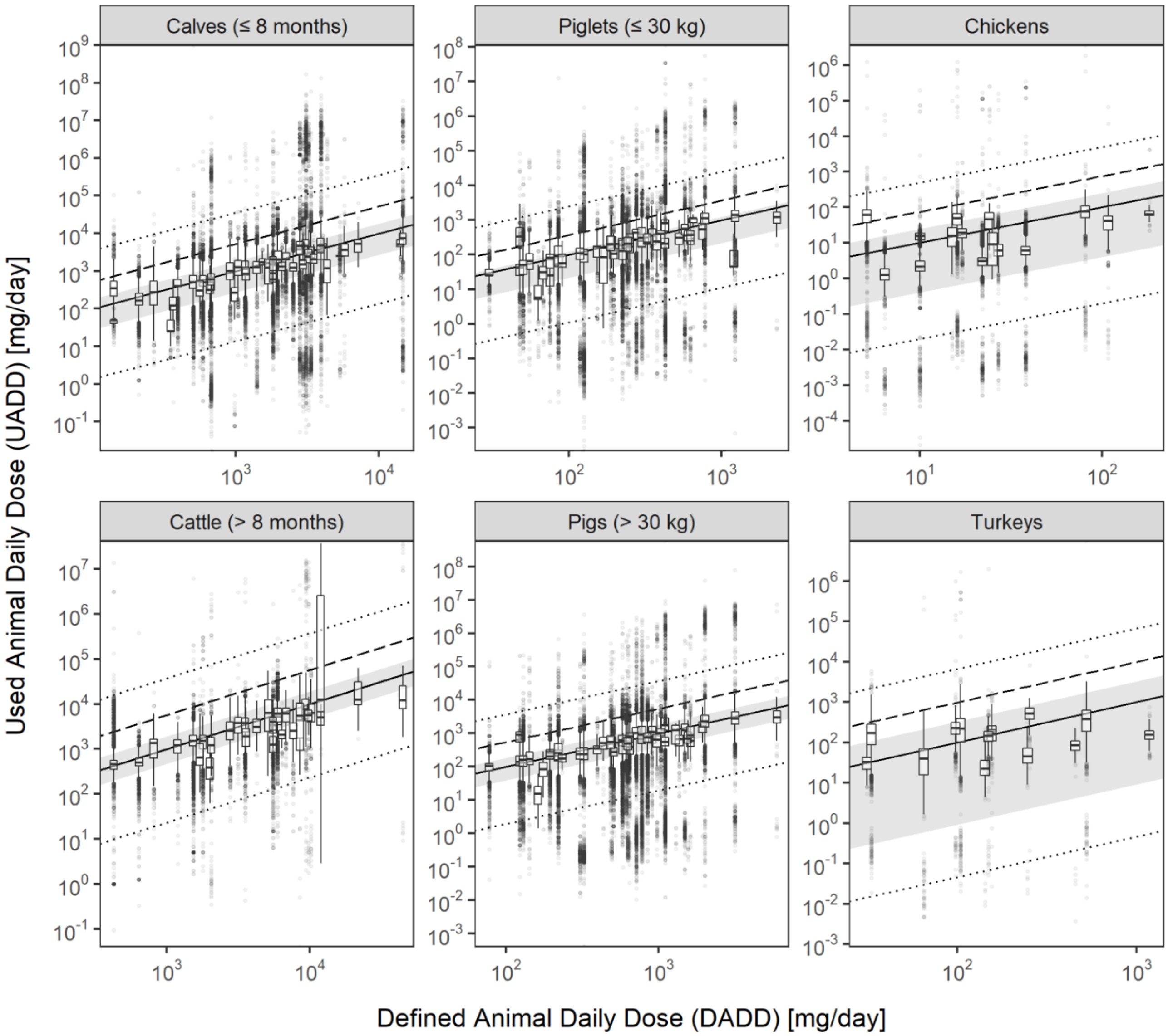

Supplementary Figure 1. Used Animal Daily Dose vs. Defined Animal Daily Dose (before removal of outliers). For each active ingredient/pharmaceutical characteristics, the distribution of UADD values is presented as a boxplot. Regions shaded in gray show UADD ranges that can be explained by animal weights (between the minimum and maximum weights according to values tabulated in 3). Solid diagonal lines show where both animal daily doses are the same (i.e. ADDR = 1). Dashed diagonal lines indicate three times the maximum weight (as used in plausibility checks for all active ingredients except colistin in 2). Dotted diagonal lines indicate twenty times the maximum weight (as used in plausibility checks for colistin in 2) and one twentieth of the minimum weight, respectively. In all animal populations and for most active ingredients, bands of outliers can be observed, located approximately at either one thousand times or one thousandth of the respective median value. These

are presumably data entry errors where the wrong unit for the amount of VMP has been recorded (e.g. g instead of kg or vice versa).

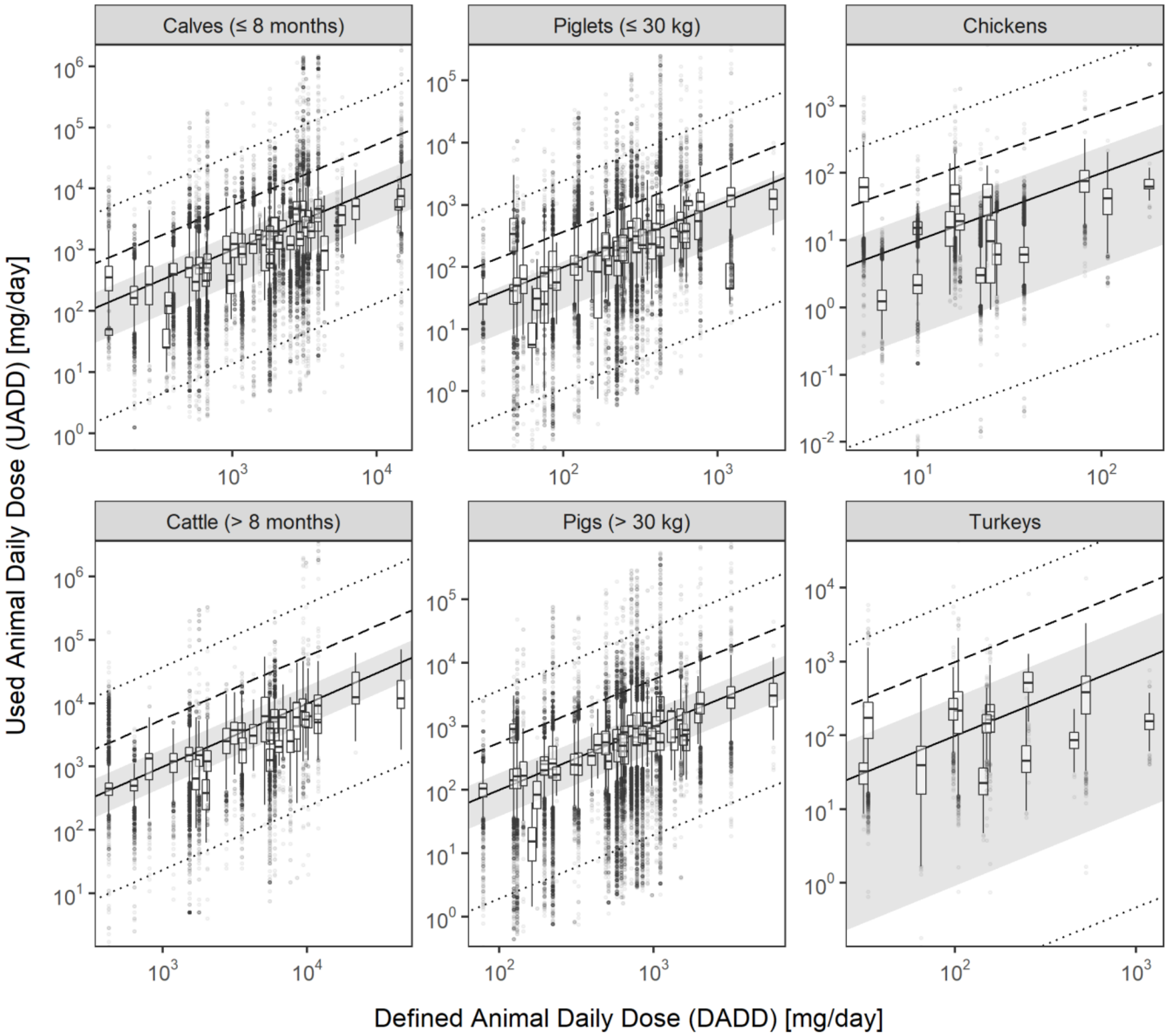

Supplementary Figure 2. Used Animal Daily Dose vs. Defined Animal Daily Dose (after removal of outliers). For each active ingredient/pharmaceutical characteristics, the distribution of UADD values is presented as a boxplot. Regions shaded in gray show UADD ranges that can be explained by animal weights (between the minimum and maximum weights according to values tabulated in 3). Solid diagonal lines show where both animal daily doses are the same (i.e. ADDR = 1). Dashed diagonal lines indicate three times the maximum weight (as used in plausibility checks for all active ingredients except colistin in 2). Dotted diagonal lines indicate twenty times the maximum weight (as used in plausibility checks for colistin in 2) and one twentieth of the minimum weight, respectively. Data classified as outliers have been removed.

3 References

1. European Medicines Agency. Defined daily doses for animals (DDDvet) and defined course doses for animals (DCDvet). Veterinary Medicines Division (2016). Report No.: EMA/224954/2016. Available from: https://www.ema.europa.eu/en/documents/other/defined-daily-doses-animals-dddvet-defined-course-doses-animals-dcdvet-european-surveillance_en.pdf.

2. Flor M, Käsbohrer A, Kaspar H, Tenhagen B-A, Wallmann J. Beiträge der Arbeitsgruppe Antibiotikaresistenz des Bundesinstituts für Risikobewertung (BfR) und des Bundesamtes für Verbraucherschutz und Lebensmittelsicherheit (BVL) zum Bericht des Bundesministeriums für Ernährung und Landwirtschaft (BMEL) über die Evaluierung des Antibiotikaminimierungskonzepts der 16. AMG-Novelle. Themenkomplex 1: Entwicklung der Antibiotikaabgabe- und -verbrauchsmengen sowie der Therapiehäufigkeit. Bonn, Germany: Bundesministerium für Ernährung und Landwirtschaft (2019). Available from: https://www.bmel.de/DE/themen/tiere/tierarzneimittel/kurzfassung16-amg-novelle.html.

3. Kuratorium für Technik und Bauwesen in der Landwirtschaft e.V. (KTBL). *Faustzahlen für die Landwirtschaft*. 15th ed. Achilles W, Anter J, Belau T, Blankenburg J, editors. Darmstadt, Germany: Kuratorium für Technik und Bauwesen in der Landwirtschaft e.V. (2018). ISBN 978-3-945088-59-3.
